# Maladjusted Host Immune Responses Induce Experimental Cerebral Malaria-Like Pathology in a Murine *Borrelia* and *Plasmodium* Co-Infection Model

**DOI:** 10.1371/journal.pone.0103295

**Published:** 2014-07-30

**Authors:** Johan Normark, Maria Nelson, Patrik Engström, Marie Andersson, Rafael Björk, Thomas Moritz, Anna Fahlgren, Sven Bergström

**Affiliations:** 1 Department of Molecular Biology, Umeå University, Umeå, Sweden; 2 Laboratory for Molecular Infection Medicine Sweden (MIMS), Umeå University, Umeå, Sweden; 3 Umeå Center for Microbial Research, Umeå University, Umeå, Sweden; 4 Division of Infectious diseases, Department of Clinical Microbiology, Umeå University, Umeå, Sweden; 5 Department of Molecular and Cell Biology, University of California, Berkeley, California, United States of America; 6 Department of Mathematics and Mathematical Statistics, Umeå University, Umeå, Sweden; 7 Umeå Plant Science Centre (UPSC), Swedish University of Agricultural Sciences (SLU), Umeå, Sweden; University of Toledo School of Medicine, United States of America

## Abstract

In the *Plasmodium* infected host, a balance between pro- and anti-inflammatory responses is required to clear the parasites without inducing major host pathology. Clinical reports suggest that bacterial infection in conjunction with malaria aggravates disease and raises both mortality and morbidity in these patients. In this study, we investigated the immune responses in BALB/c mice, co-infected with *Plasmodium berghei* NK65 parasites and the relapsing fever bacterium *Borrelia duttonii.* In contrast to single infections, we identified in the co-infected mice a reduction of L-Arginine levels in the serum. It indicated diminished bioavailability of NO, which argued for a dysfunctional endothelium. Consistent with this, we observed increased sequestration of CD8+ cells in the brain as well over expression of ICAM-1 and VCAM by brain endothelial cells. Co-infected mice further showed an increased inflammatory response through IL-1β and TNF-α, as well as inability to down regulate the same through IL-10. In addition we found loss of synchronicity of pro- and anti-inflammatory signals seen in dendritic cells and macrophages, as well as increased numbers of regulatory T-cells. Our study shows that a situation mimicking experimental cerebral malaria (ECM) is induced in co-infected mice due to loss of timing and control over regulatory mechanisms in antigen presenting cells.

## Introduction

Concomitant infections by vector borne pathogens occur at high frequencies in sub-Saharan Africa [1], [2]. However, the impact on human health is poorly understood. Evidence indicates that bacterial infection in conjunction with malaria aggravates disease and significantly raises both mortality and morbidity in these patients [3], [4]. Therefore, treatment recommendations for malaria include a regimen of broad spectrum antibiotics [5].

Infection by *Plasmodium spp* parasites is still one of the primary contributors to childhood mortality and obstetric complications in the developing world [6]. A delicate balance between pro- and anti-inflammatory responses is required for clearing of *Plasmodium* parasites without the induction of major host pathology, suggesting that the timing and the intensity of different types of responses determine the outcome of infection. The normal course of events following a malaria infection includes an acute and a chronic phase. During the acute phase, CD4+ T_H_1 activation resulting in induction of IFN-γ is required to control the initial high parasitemia [7], [8]. The chronic phase that follows is characterized by a combination of T_H_1 and T_H_2 responses involving neutralizing antibodies [9]. *Plasmodium* has been shown to impair antigen presentation and maturation of dendritic cells (DC) during infection which ultimately lead to attenuation of specific humoral responses [10]–[13]. In addition, *Plasmodium* infection induces differentiation of regulatory T cells (Tregs) resulting in a general immunosuppression [14]–[16]. Cerebral malaria is also characterized by an abnormal aggregation of CD8+ cytotoxic T-cells in the brain microvasculature. These cells are described to sequester and bind to activated endothelium in the central nervous system [17]. It has been shown that IFN-γ-driven processes are involved in this phenomenon [18] and that IFN-γ-producing CD4+ cells promote the development of experimental cerebral malaria (ECM) in murine systems [19]. Nitric oxide (NO), produced by phagocytes, has a role in controlling the pathogenesis during *Plasmodium* infection [20]. NO also has an important role in keeping the vascular tone and blood flow when secreted by endothelial cells. Children with severe cerebral malaria display a decreased level of NO production together with decreased levels of L-arginine, the precursor for synthesis of NO [21]. One consequence of dysfunctional endothelium might be increased adhesion-receptor expression on endothelial cells, leading to increased sequestration of infected erythrocytes in post capillary venules throughout the host organism [22].

Relapsing fever (RF) is caused by a vector borne, Gram negative, *Borrelia* spirochaete. The pathogen enters the host through a feeding *Ornithodoros spp* soft-bodied tick and gives rise to multiple waves of bacteremia and concurrent fever. Relapsing fever is by itself exceedingly virulent in the human host [23] and the clinical picture is very reminiscent of malaria and includes hepatosplenomegaly, neurological deficits and anemia. If left untreated, patients may present with relapsing-remitting fever over the course of several months. [23], [24]. A substantial number of patients who suffer from RF are misdiagnosed with malaria due to similar manifestation and geographic distribution of the two ailments [24]–[26]. The RF strategy for immune evasion relies on sequential expression of variable surface antigens, termed variable major proteins (vmp), on the surface of the bacteria [27], [28]. Work done in the new world RF spirochetes *B. hermsii* and *B. turicatae* show that T cell independent B-cell responses are sufficient to control bacteremia in mice [29], [30], where efficient IgM secretion is essential [31], [32]. The responses rely primarily on TLR-2 signaling [33]. Although humoral responses are necessary for the resolution of bacteremia, other non-humoral immune responses such as phagocytic cell function is incessantly important in controlling spirochaetemia in old world RF *B. duttonii* infection regardless of T and B-cell function [34], however, the underlying mechanisms have not been further investigated.

Our interest in the patho-physiological mechanisms behind the phenomenon of *Plasmodium*-bacterial concomitant infections prompted us to further elucidate the immunological mechanisms of this entity. In a previous study we developed an *in vivo* co-infection model [35]. BALB/c mice were co-infected with the murine *Plasmodium* parasite *P. berghei* NK65 and *B. duttonii* (1120K3). We observed that concomitantly infected mice were severely afflicted and that mortality was markedly increased compared to the single infected animals. Furthermore, we detected a shift of immune responses from T_H_2 to T_H_1 dominance, where the animals lost control over the *Borrelia* infection. The overall early symptoms were very similar to ECM otherwise seen in the *P. berghei* ANKA-C57BL6 mouse model [36]. We concluded that the immunopathology seen in cerebral malaria can be induced in an otherwise unsusceptible model by introducing a second priming infection, such as RF *Borrelia*. This stimulatory effect would as such also be applicable in cases of plasmodia infection in humans. Therefore, we conducted a study where we again applied the *P. berghei-B. duttonii* co-infection model in order to elucidate the dependencies of the deregulated immune response. Taken together, in this study we could show that the augmented infection response and pathological development induced in co-infected mice are due to loss of timing of immunological responses. In addition there is also a pronounced loss of regulatory control mechanisms in the antigen presenting cells.

## Materials and Methods

### Ethical statement

Female BALB/c mice were housed and procedures were performed in accordance to the regulations of the Swedish Board of Agriculture for the care and use of experimental animals (LSFS 1988∶45) and approved by the Laboratory Animal Ethical Committee in Umeå, project license number A44-11.

### Animals and infectious agents

Groups of six or ten BALB/c mice were injected intravenously with 1×10^7^ blood stage *P. berghei* NK65 parasites and/or were injected subcutaneously with 1×10^5^ *B. duttonii* 1120K3 bacteria to achieve single or concomitant infections. Mice were monitored daily, and clinical experimental cerebral malaria (ECM) evaluated. The Rapid Murine Coma and Behavior Scale [37] was used for detailed scoring of animal fitness and presence of symptoms. For termination purposes, clinical ECM scores were defined by the presentation of the following signs: ruffled fur, hunching, wobbly gait, limb paralysis, convulsions and coma. Animals with severe ECM (accumulative scores ≥4) were sacrificed either by CO_2_ asphyxiation or by lethal cardiac puncture in Ketamine/Medetomidine narcosis according to ethical guidelines. The day of death was deemed to be the following day when applicable. Mice were infected as previously described with either or *B. duttonii* clonal strain 1120 K3 and *P. berghei* NK65 [35]. Parasitemia was monitored by counting infected erythrocytes in Giemsa-stained thin smears of tail blood and bacteremia was monitored by counting cells in tail blood in a Petroff-Hausser chamber. Parasitemia and bacteremia scores are displayed in Figure S1.

### Multiplex quantification of cytokines

EDTA serum samples was isolated from blood obtained by cardiac puncture on days indicated post infection. Cytokine concentrations were determined using a Bio-Rad Luminex Mouse cytokine kit according to the manufacturer’s instructions. The fluorescent beads were analyzed on a BioPlex 200 system.

### Preparation of tissue single cell suspensions

Anesthetized mice were exsanguinated through cardiac puncture through the left ventricle and the circulatory system was perfused with ice cold PBS. Spleens or brains were dissected and kept on ice in PBS. The spleen tissue was then homogenized through a 100 µm cell strainer while kept in phosphate-buffered saline supplemented with 2% (v/v) fetal bovine serum (wash buffer). The cell suspension was overlaid on a Ficholl-Paque (GE Healthcare) column and centrifuged at 693×*g* for 30 minutes at 4°C. The mononuclear cell layer was secured and then washed three times in wash buffer by centrifugation in 514×*g* for 5 minutes at 4°C. The pellet was resuspended and the cells were subsequently counted. Brain mononuclear cells were isolated according to Pino et al [38]. Mice were sacrificed as above and the circulatory system perfused with ice cold 1× HBSS. The tissues were homogenized in a Dounce homogenizer containing RPMI, suspended into Percoll to make a 30% Percoll solution. This was layered on top of a 70% Percoll in 1× HBSS w/o Ca^++^ and Mg^++^ solution and centrifuged 30 min at 500×*g* at 18°C. The 70%–30% interphase was secured and added to fresh 1× HBSS and centrifuged again 7 min at 500×*g* at 18°C. The cell pellet was then resuspended in 1 ml Tryphane blue cell staining buffer and washed once more in 1× HBSS and then counted.

### Flow cytometry

Cells were suspended in FACS buffer (PBS supplemented with 1% FCS and 0.1% sodium azide) at a concentration of 2×10^6^ cells/ml. Fifty microliters of the appropriately diluted antibody were added to 250 µl of the cell suspensions. After incubation at 4°C for 30 min, unbound antibodies were removed by three washing steps, including addition of 750 µl FACS buffer and centrifugation at 300×*g* for 2 min at 4°C. Flow cytometric surface expression analyses were performed with anti-mouse CD3, CD4, CD8, CD11c, CD25, CD83, CD86, F4/80, MHCI, or MHCII Abs (eBioscience) as Alexa 488, allophycocyanin, or PE conjugates. 7-Aminoactinomycin D was used as dead cell control. Appropriate controls were performed to ensure the specificity of the labeling reactions including use of irrelevant isotype control immunoglobulins and omission of key reagents. Intracellular staining was performed with the Foxp3 staining kit from eBioscience (NatuTec, Frankfurt, Germany). Gating strategies are found in Figures S2 and S3. Flow cytometric analysis of stained cells was performed using a LSRII and DIVA software (Becton Dickinson, Heidelberg, Germany) as well as FlowJo (Tree Star Inc, Ashland, OR).

### Analysis of Arginine and ADMA

For analyses of Arginine and ADMA 25 µl of serum was extracted with 225 µl of 90% MeOH, including ^13^C_6_^15^N_3_-arginine as internal standard. The extract was mixed for 10 sec, kept on ice for 10 min, and then vigorously extracted at a frequency of 30 Hz for 3 min using a MM301 vibration Mill (Retsch GmbH & Co. KG, Haan, Germany). After 120 min on ice, the samples were centrifuged at 19 600 g for 10 min at 4°C and 50 µL of the extract was transferred to a vial and evaporated to dryness. The dried extracts were stored in −80°C until derivatization and analysis. The samples were subsequently derivatized using 6-aminoquinolyl-N-succhinimidyl carbamate (ACQ) according to the manufacturer’s protocol (AccQ•Tag kit, Waters, Milford, MA, USA). In brief the dried extracts were resuspended in 20 µL of 20 mM HCl, 60 µL of AccQ•Tag Ultra borate buffer was added to each vial. Finally 20 µL of the freshly prepared AccQ•Tag derivatization solution was added and the sample was immediately vortex for 10 s. After mixing the samples were standing for 30 minutes at room temperature followed by 10 minutes at 55°C.

Analytes were separated using a HP 1200 LC system from Agilent Technologies (Waldbronn, Germany), consisting of a G1379B online vacuum degasser, G1312B binary pump, G1316B thermostated column compartment and G1367D autosampler with G1330B autosampler thermostat. A 2 µL aliquot of the sample was injected onto a 2.1×50 mm, 1.7 µm HPLC column (Kinetex, Phenomenex) held at 55°C in a column oven. The gradient elution buffers were A (H_2_O, 0.1% formic acid) and B (acetonitrile, 0.1% formic acid), and the flow-rate was 300 µl min^−1^. The initial condition was 0.1% B, from 0.54 to 8 minutes the B eluent was linearly increased from 0.1% to 6%. From 8 minutes B was linearly increased to reach 50% at 10 minutes, and kept there for 1 minute. From 11 to 12 minutes the column was returned to its initial conditions (0.1% B), the column was equilibrated for 3 minutes before injection of the subsequent sample.

Arginine and ADMA were detected with an Agilent 6460 triple quadrupole mass spectrometer equipped with a jet stream electrospray source operating in positive ion mode. The jet-stream gas temperature was 325°C with a gas flow of 10 L hr^−1^, and a sheath gas temperature equal 400°C and flow of 12 L hr^−1^; nebulizer pressure was set to 25 psi. The ion spray voltage was set at 4 kV for positive ion mode, nozzle voltage was set to 0 V. The analyses were performed in multiple-reaction-monitoring (MRM) mode where the fragmentation conditions for the analytes were optimized using MassHunter MS Optimizer software (Agilent Technologies). Dwell time was 50 ms, and nitrogen was used as collision gas.

The LC/MS analysis was controled by the MassHunter software v 2.00 and data processing by the MassHunter Quantitation software v 4.00 (Agilent, USA).

### Immunofluorescence and histology

Cryo-sectioned brain tissue, mounted on Superfrost microscope slides (Menzel-Gläzer), was fixed in 4% paraformaldehyde (PFA) and subsequently incubated in 0.1 M glycine to reduce auto-fluorescence. Next, to reduce non-specific binding of primary antibodies, samples were incubated in 1% BSA and 5% goat and rabbit-serum for at least one hour. The tissue sections were stained for CD8+ cell presence with a primary anti-CD8α antibody (rat anti-mouse, BD Pharmingen) and a primary anti-CD3 antibody (guinea-pig anti-mouse, Aviva Systems Biology). An Alexa 594 rabbit anti-rat antibody was used to detect CD8α and an Alexa 488 goat anti guinea-pig antibody was used to detect CD3 (both secondary antibodies from Invitrogen). DNA was stained with 200 nM 4′,6-diamidino-2-phenylindole (DAPI). Samples without primary antibodies were used to define the background. Tissue sections treated in the same way with PFA and glycine were also stained for the presence of ICAM and VCAM. Endogenous peroxidase activity was blocked by incubation in 0.03% H_2_O_2_ for 30 min. Sections were incubated with avidin and biotin (Blocking kit, Vector Laboratories Inc, Burlingame) for 10 min, respectively, followed by 0.5% BSA for 30 min. Incubations with primary antibodies proceeded overnight at 4°C on sequential sections. Sections incubated with rat monoclonal antibodies, ICAM1 (rat anti-mouse CD54, eBioscience) and VCAM-1 (rat anti-mouse CD106, eBioscience) were incubated with biotinylated rabbit anti-rat antibodies (DAKO Corp.) for 1 h. StreptABComplex/HRP (DAKO Corp.) was used according to the manufacturer’s recommendations for amplification of signals, and developed using DAB chromogen tablets (DAKO Corp.). Methyl Green (Sigma-Aldrich) was used as counterstain. Brains were also parafinized, sectioned and mounted on glass slides. The sections were then deparafinized, hydrated and stained with Hematoxylin/Eosin. Images were obtained by confocal laser scanning or brightfield microscopy (Nikon Eclipse C1 plus) and processed by Adobe Photoshop software (Adobe Systems Inc.).

### Statistical analysis

Experiments were performed independently in duplicates or triplicates and means were calculated for each time-point. Statistical analysis was performed using the R statistical software environment and Sigma Plot (Systat Software Inc). P<0.05 was considered significant.

## Results

### Endothelial dysfunction is exacerbated in concomitant infection

**Figure 1:**
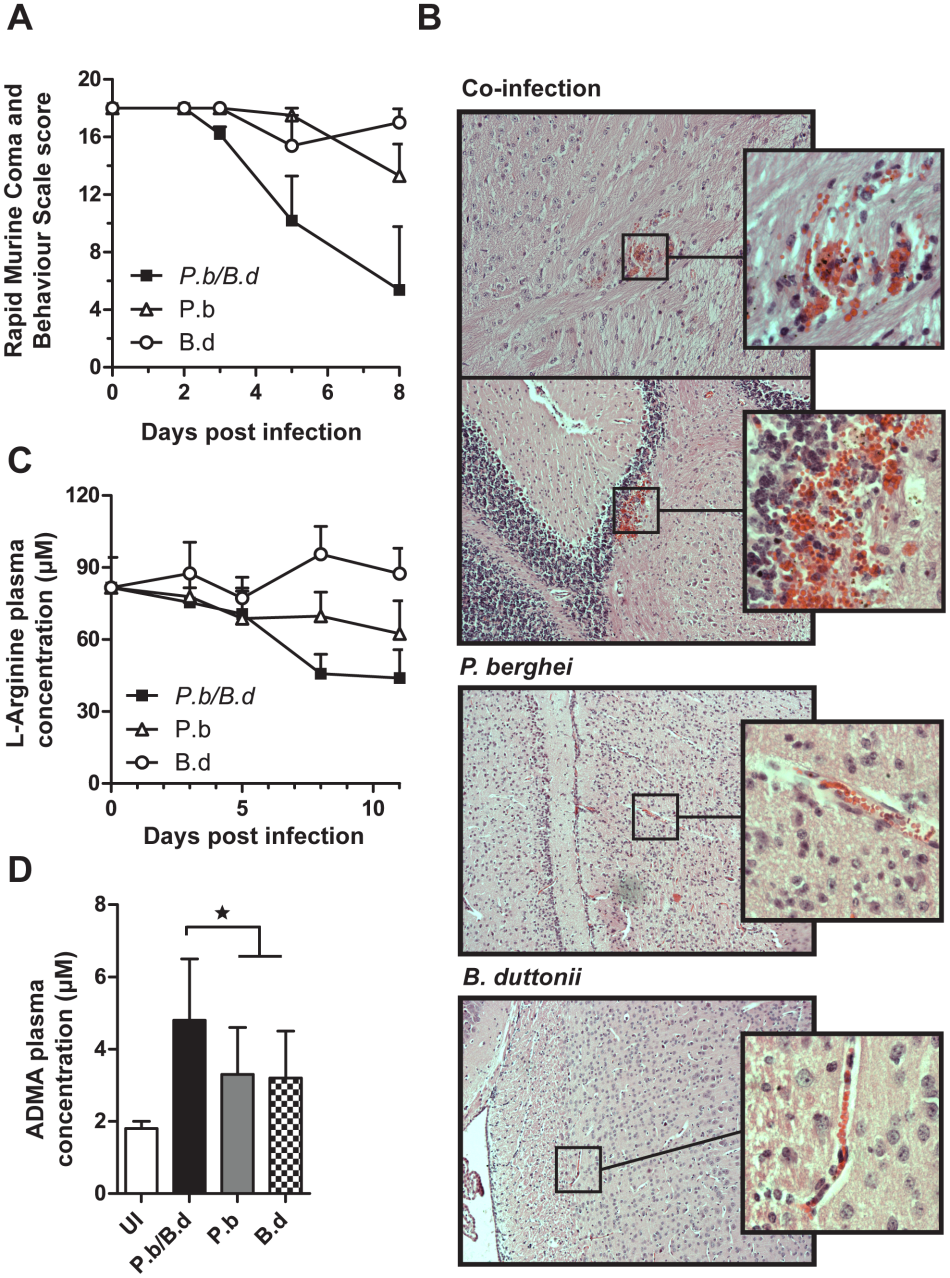
Co-infection causes brain endothelial damage and reduced L-Arginine levels. (A) Mice were monitored and scored for clinical signs of ECM using the rapid murine coma and behavior scale (RMCBS) (N = 6 per group; values are means ±SD, experiment performed in duplicate) (B) Images of brain tissue from mice co- and single infected day 5 p.i. stained with Hematoxylin-Eosin. (C) Plasma was sampled from infected mice, and the concentration of L-Arginine in plasma is shown. (▪) indicates mice infected with both *P. berghei* and *B. duttonii*, (○) *B. duttonii* infected animals and (Δ) mice infected with *P. berghei* (N = 10 per group; values are means ±SD, experiment performed in duplicate). (D) Concentration of N^G^, N^G^-dimethylarginine (ADMA) in plasma on day 5 p.i. UI is an acronym for uninfected mice. *P. berghei*/*B. duttonii* indicates mixed infection, P.b mice infected with *P. berghei* and B.d animals infected with B. duttonii. * indicates a significant difference of P<0.05 tested with Students t-test (N = 10 per group; values are means ±SD, experiment performed in duplicate).

The clinical pathology, reminiscent of ECM seen in co-infected mice in our previous trials [35], prompted us to investigate a potential dysfunction of the endothelium. BALB/c mice were infected simultaneously or separately with *P. berghei* and *B. duttonii* infectious agents. Clinical scoring of the animals using the Rapid Murine Coma and Behavior Scale [37] revealed successive accumulation of neuropathological symptoms including ataxia, tremor and loss of motor function. The clinical score was significantly lower in the co-infected group day 8 post infection (p.i.) compared to *P. berghei* infected animals (t-test, P = 0.003). The mice scored well into the range seen in animals afflicted by ECM (Figure 1A). In addition to this we could see the presence of ring hemorrhages around vessels in the brains of co-infected mice. The hemorrhages were found both in the cerebrum and cerebellum, primarily located in the junction regions between white and gray matter, but could also occur scattered in the white matter parenchyma (Figure 1B), These hemorrhages were absent in the single infected mice. L-Arginine is a key amino acid in several metabolic pathways including synthesis of NO, and is considered of main importance during severe malaria [20]. Using mass spectrometry, we observed that the co-infected mice displayed significantly lower concentrations of L-Arginine in plasma in the latter part of the infection compared with both single infections (Figure 1C), indicating low levels of available NO. We also measured amounts of N^G^, N^G^-dimethylarginine (ADMA) in mouse plasma (Figure 1D). ADMA is a non-reversible inhibitor of NO synthetase implicated in endothelial damage on many different levels [39]. We observed that during the mid-infection time point (day 5 p.i.), ADMA concentrations were significantly elevated in the co-infected animals compared to the single infections (P = 0.028 and 0.014, co-infected versus *P. berghei* and *B. duttonii* respectively, t-tests, N = 10 in each group), further indicating that production of NO was inhibited. Early in the infection there was no significant difference of ADMA levels between the co- and single infected subjects. In contrast, in the latter part of infection the inter-individual variation was very high, where certain *Borrelia* infected subjects, co-infected or not (N = 4 out of 19) increased their plasmatic ADMA concentration by a factor of >10. This correlated to high levels of bacteremia (Fisher exact test, P = 0.025), with high bacteremia levels defined as ≥5×10^6^ bacteria/ml blood (Figure S1). Thus, low NO-bioavailability in co-infected mice leads to an increase of cell-adhesion molecules on the endothelial lining and possibly vascular leak. The effect is correlated to the bacterial load, thus it appears that the bacteria are responsible for this effect in co-infected mice.

### Co-infection induces intra cerebral immune-pathology associated to ECM

**Figure 2:**
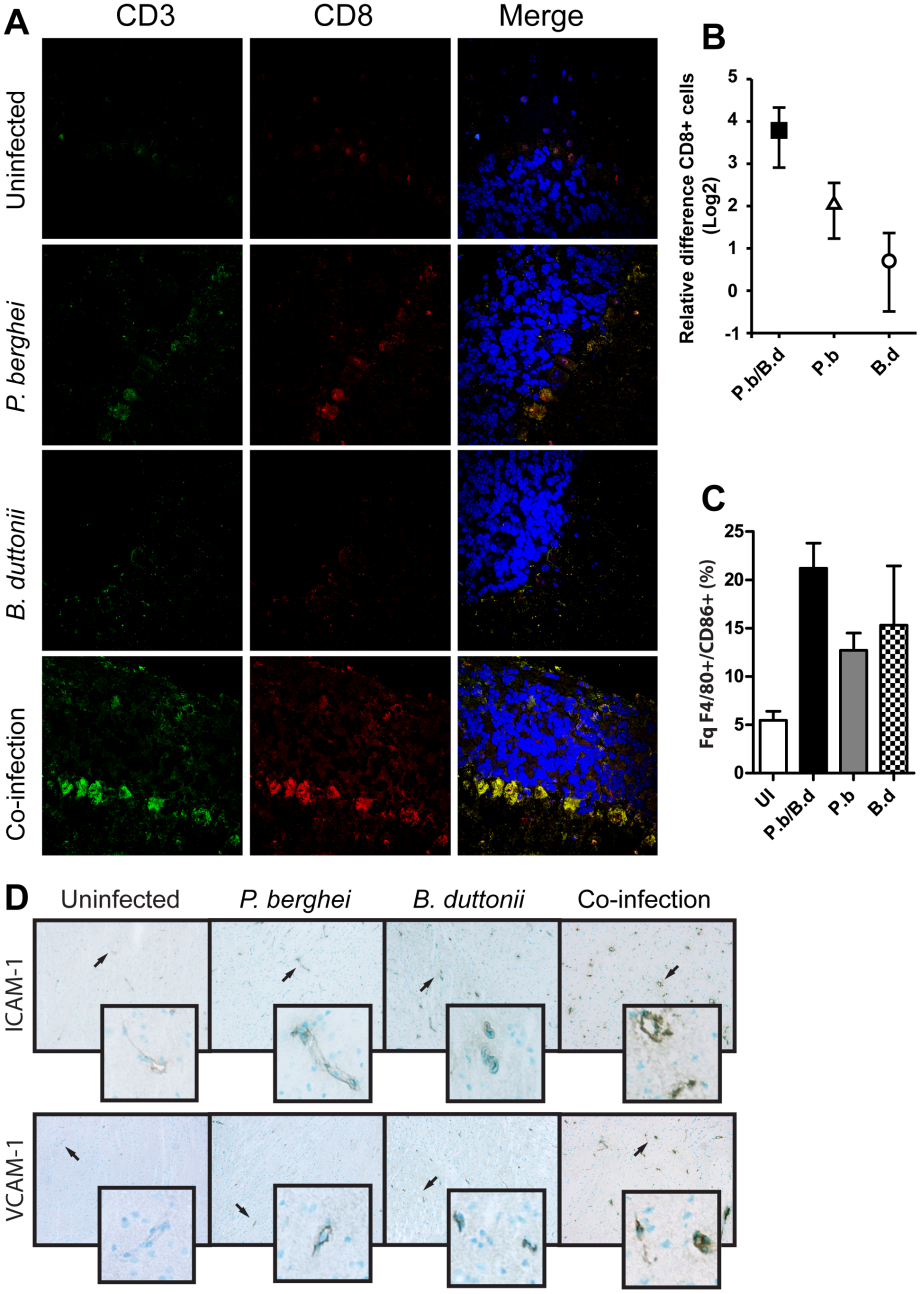
The brain in co-infected mice contain high amounts of CD8+ cells and activated MΦs. Brains from mice infected with *P. berghei*, *B. duttonii* or both as per M&M were collected day 5 p.i. (N = 6 per group), experiments performed in duplicate (A) Cryo-sectioned tissue double stained for CD3 and CD8 positive cells and DNA visualized with DAPI. Representative regions in the frontal cortex were imaged by confocal laser microscopy. Tissue stained in absence of primary CD3 and CD8 antibodies served as negative controls. (B) The relative fold-change differences in absolute numbers of CD8+ cells. (C) The percentage frequency of CD86+ MΦs in the total pool of F4/80+ cells extracted from the brains (N = 6 per group, experiment performed in duplicate). (D) Cryo-sectioned brain tissue, immuno-stained for ICAM and VCAM.

Since we found the endothelium to be dysfunctional in co-infected animals, probably due to low NO bioavailability, we sought to investigate if CD8+ cell sequestration in the brain was affected. We analyzed presence of CD3+CD8+ T-lymphocytes in the cerebral circulation by immunofluorescence histochemistry on day 5 p.i. We observed that co-infected brains displayed a higher number of CD3+CD8+ cells than brains from single infected animals (Figure 2A), with the cells lying adjacent to each other and to vessel walls. We also enumerated the total amount of CD8+ T-cells in the brain and found that co-infected mice displayed a fourfold increase thereof compared to the uninfected mice (Figure 2B, t-test, P<0.001, Power of performed test with alpha = 0.050∶1.000).

The intravascular accumulation of macrophages in the brain has also long been recognized both in mice and humans with cerebral malaria symptoms [40]. These macrophages are active and are an abundant source of pro inflammatory cytokines such as TNF-α. We therefore explored the possibility of over-activated MΦs being present in the brain in co-infected mice. By detection of CD86 levels on brain MΦs we found that all three modalities of infection displayed a rise in MΦ activation compared to control (Figure 2C). MΦs from co-infected mice showed highest levels of expression, significantly greater than single *P. berghei* ditto (P<0.001, t-test, Power of performed test with alpha = 0.050∶1.000) but not significantly more than *B. duttonii* single infected mice (Figure 2C).

An impressive body of evidence shows that cerebral malaria is associated not only to CD8 T-cell sequestration in the brain microvasculature, but also sequestration of infected red blood cells to the same. The up-regulation of vascular cell adhesion molecules is a feature of cerebral malaria [41] in parallel to reduced bioavailability to NO. While Intercellular adhesion molecule-1 (ICAM-1) overexpression has been shown to be independent of ECM [42], the phenomenon can be appreciated in the case of human *B. duttonii* and *P. falciparum* co-infection as a pro-sequestration driven process. We therefore used immunohistochemistry to identify ICAM-1 and vascular cell adhesion molecule-1 (VCAM-1) expression in endothelium (Figure 2D). Hence, we could see that the brain endothelium in all co-infected mice stained more intensely for both ICAM-1 and VCAM compared to all other infection modalities under the same conditions. The data suggest that co-infection indeed triggers hallmark processes found in ECM.

### *Plasmodium* and RF *Borrelia* co-infection induces a temporal shift in IL-10 signaling and regulatory T-cell populations

**Figure 3:**
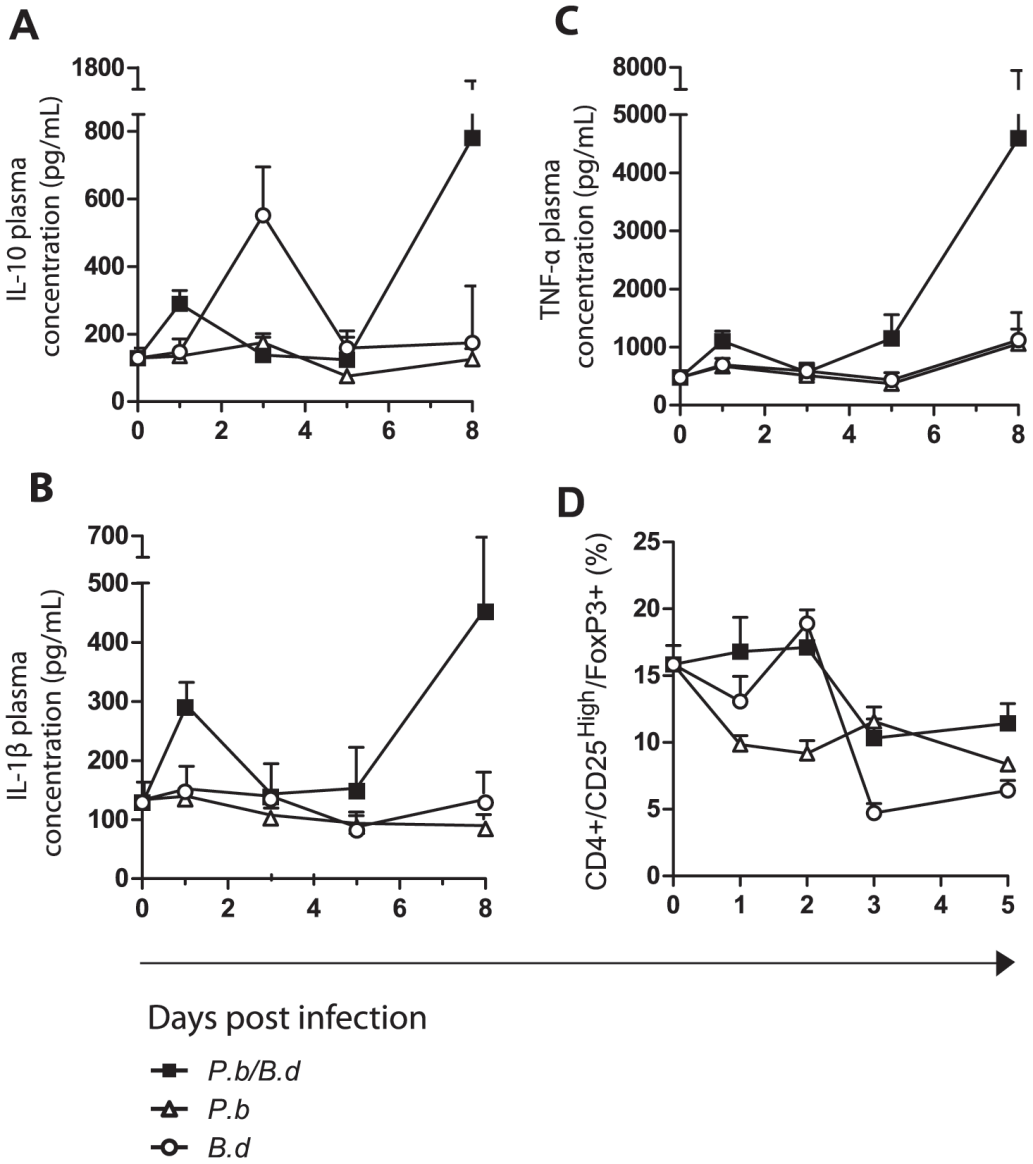
Co-infection causes conflicting cytokine signaling and persisting nTreg populations. Mice were injected with 1×10^7^ *P. berghei* NK65-infected RBCs and/or 1×10^5^ *B. duttonii* 1120K3 bacteria, and then sacrificed at indicated time points (N = 6 per group). Cytokine level experiments performed in triplicate, nTreg experiment performed in duplicate. Plasma levels of cytokines were measured with multiplex ELISA (A) Plasma IL-10 concentration (pg/mL) (B) Plasma IL-1β concentration (pg/mL) (C) Plasma TNF-α concentration (pg/mL) (D) The percentage fraction of CD4+CD25^high^FoxP3+ cells, in the total CD4+ cell pool in the spleen determined with flow cytometry. (▪) Represents mice infected with both *P. berghei* and *B. duttonii*, (○) indicates animals infected with *B. duttonii* and (Δ) mice infected with *P. berghei*.

The signs of inflammation induced pathology seen in the brain motivated us to analyze the early inhibitory and excitatory mechanisms that control the cellular immune responses to infection. We first analyzed levels of IL-10, an anti-inflammatory cytokine that down-regulates the expression of MHC class II molecules, co-stimulatory, and adhesion molecules. Single infection with *P. berghei* did not elicit an elevation of IL-10 during the course of the infection (Figure 3A), and pro-inflammatory cytokines (IL-1β, TNF-α and IFN-γ) were not elevated in plasma (Figures 3B, 3C and [35]). In comparison, single infection with *B. duttonii* caused a peak in IL-10 levels day 3 p.i. In co-infected mice, the IL-10 levels were significantly increased compared to uninfected control early (day 1 p.i.), and late (day 8 p.i.) (Wilcoxon rank sum test, P = 0.026 and 0.004, *B. duttonii* and *P. berghei* respectively) (Figure 3A, Table S1). Moreover, this coincided with a burst of the pro-inflammatory cytokines IL-1β and TNF-α in early and late stages of infection (day 8 p.i.). Pro-inflammatory cytokines were induced at higher levels earlier in co-infected mice compared with both single infections (day 1 p.i.) (Figures 3B and 3C, Table S1).

Because of the apparent signs of aberrant cytokine responses in peripheral blood, we quantified the relative frequencies of natural regulatory T-cells (nTregs) (CD4+CD25^high^FoxP3+ cells) in spleen tissue of the infected animals (Figure 3D). The *B. duttonii* infected mice displayed a dynamic number of nTregs, while *P. berghei-*infected mice exhibited an enduring reduction of the nTreg pool. In co-infected mice we observed that the frequencies of nTregs in the total pool of CD4+ cells were significantly higher early (day 1 p.i.) compared to both of the single modalities (P = 0.026 and 0.002 *B. duttonii* and *P. berghei* respectively, Wilcoxon rank sum test, Table S1). In later stages (day 3 p.i. and subsequently) the nTreg frequencies are reduced. These data indicate that nTreg numbers are not affected early by the co-infection but at later stages *B. duttonii* suppress nTreg numbers to maintain T_H_1 dominance.

### High MHCII expression and impaired activation of dendritic cells correlates with increased bacterial load in co-infected animals

**Figure 4:**
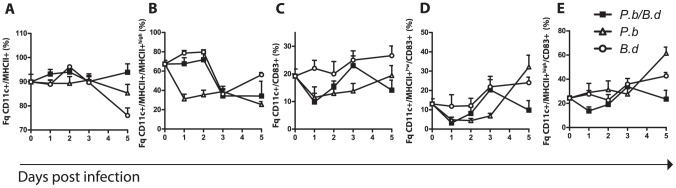
Dendritic cell responses are delayed during co-infection. Spleen mononuclear cells (SMC) were extracted on consecutive days from single and co-infected mice. Splenocytes were stained and acquired by flow cytometry (N = 6 per group), experiment performed in duplicate. (A) The percentage fraction of MHCII expressing cells in the CD11c+ population of SMCs. (B) The proportion of CD11c+MHCII+ DCs expressing MHCII to a high extent (MHCII+^high^). (C) The fraction of CD11c+ DCs expressing the activation marker CD83. (D) The fraction of CD11c+MHCII+^low^ cells expressing CD83. (E) The fraction of CD11c+MHCII+^high^ cells expressing CD83, (▪) indicates mice infected with both *P. berghei* and *B. duttonii*, (○) *B. duttonii* infected animals, and (Δ) mice infected with *P. berghei*.

**Figure 5:**
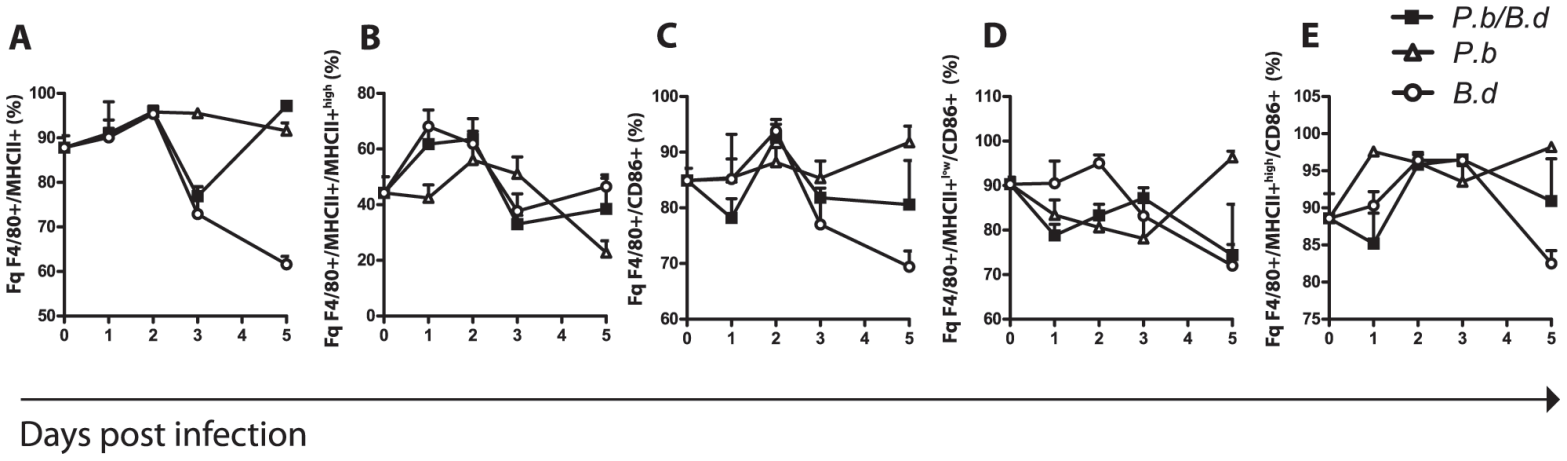
Dynamic MHCII presentation and delayed activation of MΦs during co-infection. Spleen mononuclear cells were extracted on consecutive days from single and co-infected mice. They were stained and counted in a LCRII flow cytometer (N = 6 per group) experiment performed in duplicate. (A) The percentage fraction of MHCII expressing cells in the F4/80+ population of SMCs. (B) The proportion of F4/80+MHCII+ MΦs expressing MHCII to a high extent (MHCII+^high^). (C) The fraction of CD11c+ DCs expressing the activation marker CD86. (D) The fraction of F4/80+MHCII+^low^ cells expressing CD86. (E) The fraction of F4/80+MHCII+^high^ cells expressing CD86, (▪) indicates mice infected with both *P. berghei* and *B. duttonii*, (○) *B. duttonii* infected animals, and (Δ) mice infected with *P. berghei*.

In order to unravel the origin of the affected peripheral cytokine levels during co-infection, the antigen presentation potential and activation of immune regulating cells in the spleen were evaluated and quantified. Total spleen cells from single *P. berghei*, single *B. duttonii* and co-infected mice (N = 6 from each infection type and time point) were isolated at day 0–5 p.i. Dendritic cells (DCs) and macrophages (MΦ) were analyzed for expression of the cells surface markers MHCI and MHCII, and the co-stimulatory molecules CD83 and CD86 (Figures 4 and 5). We found that the different modes and duration of infection greatly influenced the mean fluorescence intensity (MFI) of MHCII+ on both DCs and MΦs (the amount of MHCII molecules present on the cell surface is directly proportional to the MFI). In both single- and co-infected tissues, we detected and gated two distinct populations with high (MHCII+^high^) and low (MHCII+^low^) expression of MHCII (Figures S2 and S3) reflecting the state of maturity of the DCs since mature DCs present more MHCII molecules on the surface than their immature counterparts [43], [44]. MHCII+^high^ expressing DCs presumably efficiently propagate signal to recipient T-cell effectors. Consistent with data from other groups [13] [12] we observed that single *P. berghei* infection showed reduced MHCII expression by DCs (MHCII in total, Figure 4A, day 5 p.i. and MHCII+^high^ Figure 4B, all time points p.i. significantly lower compared to uninfected control, Table S2), while DCs from *B. duttonii* infected mice displayed an oscillating MHCII+^high^ expression curve (Figure 4B), from high (day 1 and 2 p.i.) to low (day 3 p.i.). Dendritic cells from the co-infected animals showed a general increase in MHCII expression on the surface day 2 p.i. (Wilcoxon rank sum test, P = 0.004) and otherwise no MHCII reduction (Figure 4A). The fraction of CD11c+ MHCII+^high^ cells (DCs) remained on the same level as DCs from the uninfected animals day 1–2 p.i., after which the levels dropped to levels observed in *B. duttonii* infected animals (Figure 4B). In the case of MHCI expression it was present at high levels in 98±0.5% of all CD11c+ cells by (N = 6 animals in each group) and no relative reduction of MHCI expression was seen in any of the infection modalities (data not shown).

Dendritic cell activation is required in order to propagate signal to T-cells. We observed that expression of the co-stimulatory molecule CD83 was inhibited early in *P. berghei* infections, while dendritic cells from *B. duttonii* infected animals showed a successive rise in CD83 expression as the infection progressed (Figure 4C). We could in the co-infected animals detect a significant drop in CD83 expression initially (day 1 p.i.) in the total DC pool as well as in the DCs with differing MHCII expression (Figures 4C–E, Table S2). Subsequently, the CD83 expression levels rose day 3 p.i. to that of the DCs from the *B. duttonii* infected animals, where after it fell again day 5 p.i. This was also reflected in the MFI of CD83 at each time point (Figures S4 A–C). In summary, these results show that albeit that the MHCII expression levels initially are high in co-infected animals (similar to levels in single *B. duttonii* infection), the DCs cannot activate and mature adequately. These observations correlated with increased bacterial load. Thus, impaired activation and maturation of DCs may lead to high bacteremia.

### Co-infection impairs Macrophage function through reduced activation

Since phagocyte function in general has been shown to be important to the clearance of relapsing fever spirochetes [34] we also sought to explore macrophage function in the same context as the DCs. Single *P. berghei* infected animals displayed an high overall MHCII surface expression on MΦs (Figure 5A), while the frequency of MHCII+^high^ cells peaked at day 2 p.i. and ended at low frequencies (Figure 5B, Table S3). Spleen MΦs in co-infected mice closely followed the pattern of MHCII+^high^ expression by MΦs of single *B. duttonii* infected mice with an oscillating pattern of surface expression. The peak in MHCII presentation preceded the peak of bacteremia. However, there was a pronounced difference in activation of MΦs from the co-infected mice compared to the single infections. Expression of CD86 was generally high in MΦs from *P. berghei* infected animals (Figure 5C). The MHCII+^high^ surface expression pattern of MΦs from co-infected animals was akin to *B. duttonii* infection and displayed a peak of expression day 2 p.i. (Figure 5B), corresponding to the start of the first wave of bacteremia. While MHCII+^high^ expression patterns concurred between single *B. duttonii* and co-infected animals, they displayed different levels of activation measured by the co-stimulatory molecule CD86 (Figure 5E). We could detect an initial (day 1 p.i.) fall in CD86 expression in the MHCII+^high^ co-infected animals, lower than both single infections (Wilcoxon rank sum test, P = 0.03 and 0.005, *B. duttonii* and *P. berghei* respectively). Furthermore, the co-infected spleen MΦs did not respond with an activation increase later in infection (day 5 p.i.) (Figures 5C–E, Table S3). This was mirrored more explicitly in the MFI of CD86 in the corresponding time points and infection modalities (Figures S5 A–C). MHCI presentation was also assayed in the MΦs and shown to be unaffected by any of the infections (96% ±0.7%, N = 6 in each group, data not shown). The data suggest that just like for DCs, co-infection interferes with the ability of MΦs to dynamically mature and respond to bacterial challenge.

## Discussion

The major findings of this study can be summarized as follows. In co-infected mice we show a reduced bioavailability of NO, increased sequestration of CD8+ cells as well as over expression of ICAM-1 and VCAM arguing for a dysfunctional brain endothelium in these animals. Additionally, we demonstrate increased primary inflammatory response in co-infected mice through IL-1β and TNF-α and an inability to down regulate the same through IL-10, suggesting a predominant T_H_1 response. Our study suggests that loss of control in the immune system, characterized by the loss of timing in antigen presenting cell activation, give rise to cerebral malaria-like pathology in the double infected mouse.

In co-infected mice, a mix of pro-and anti-inflammatory signals is initiated early, in contrast to single infections where the responses are concertedly pro-inflammatory the first day. Also the nTreg numbers were maintained at day 1 p.i. which coincided with early IL-10 production (day 1 p.i.). This was accompanied by a pro-inflammatory stimulus displayed as a rise of IL-1β and TNF-α in plasma. We interpret this as that a constant number of nTregs early in the infection is not sufficient to down regulate pro-inflammatory responses. In parallel, both DCs and MΦs from co-infected animals displayed initial high surface expression of MHCII while these MHCII+^high^ cells displayed a low grade of co-stimulatory markers CD83 and CD86. Thus, antigen presentation by MHCII is likely not affected. However, as co-stimulatory signals are low this would likely lead to dysfunctional T-cell activation or even T-cell anergy.

The later responses in co-infected mice, i.e. day 3 p.i. and henceforth, showed a successive rise in inflammatory stimulus through increased presence of pro-inflammatory cytokines in plasma. The antigen presenting cells in spleen tissue from co-infected mice showed intermediate MHCII+^high^ expression levels, in between what we saw in *B. duttonii* and *P. berghei* infected animals. The activation molecules were down regulated on DCs and intermediate in MΦs in co-infected mice. Hence, there are more cells presenting MHCII+^high^ in co-infected mice compared with *P. berghei* infected ones, but of these cells a lower percentage are indeed activated. Judging from our previous *in vivo* data [35] it was obvious that the response was still sufficient to control parasitemia. We believe this is likely because of more efficient phagocytosis. On the other hand this situation was deleterious to bacterial control as T_H_2 induction was too weak. The reduction of activation of the APCs inhibits efficient B-cell activation, which potentially allows efficient growth of bacteria that in turn could cause an unspecific burst of pro-inflammatory cytokines. This is enough to uphold a perpetual T_H_1 response, CD8 cell activation and high splenic clearance of parasites but also facilitates bacterial growth. In the single infected animals, a response-activation-suppression process was seen in the off and on nTreg numbers, MHCII presentation and activation-deactivation (CD86) in MΦs. We propose that the contradictory signaling events early in the response to infection initiate a cascade of T_H_1 events, promoting bacterial proliferation. Our previous hypothesis [35] suggested a shift in the T_H_1/T_H_2 cytokine balance during co-infection with predominance towards T_H_1 responses as seen in *P. berghei* infected animals.

An intense pro-inflammatory immune response promoted by IFN-γ, IL-12, and TNF-α early in a *Plasmodium* infection inhibits parasite proliferation and abrogates severe disease [8], [45]. Also, a well-timed and distinct anti-inflammatory response, represented by IL-10 and TGF-β from nTregs is likely needed to quench the initial pro-inflammatory burst [46], [47] and to promote activation of CD4+ cells and the production of anti–*Plasmodium* neutralizing antibodies [8], since T_H_1 and T_H_2 responses are mutually inhibitory [48]. Parasite-parasite co-infection has been investigated in detail previously and especially *Plasmodium* and helminthiasises have gathered much interest. It has been suggested that by creating an anti-inflammatory immune environment, helminth co-infection may dampen both protective and immunopathological responses to *Plasmodium* parasites, thus altering *Plasmodium* infection dynamics and disease severity [49]. Both synergistic effects [50], [51] and the opposite [52] have been reported in the literature. Chronic helminth infection induces T_H_2 and regulatory signaling through induction of nTreg activity [53], [54]. It decreases the risk of severe malaria disease albeit increases the chances for clinical malaria early in the infection. A similar phenomenon was observed in murine co-infections with *Trypanosoma cruz*i and *P. berghei* ANKA, where the acute symptoms and pathological signs of ECM were absent [55]. There is a lack of data regarding *Plasmodium* and acute bacterial infection pathology. Intracellular bacteria in relation to *Plasmodium* have recently gathered attention in that the hemolysis caused by the parasites impairs granulocyte mobilization. This inhibits the oxidative burst and impairs bacterial killing [56]. *Borrelia duttonii* on the other hand is an extracellular pathogen. This is the first study of its kind that in detail explores the immunological relationships between malaria and RF borreliosis.

Our work presented herein suggests that infection by *B. duttonii* in the *Plasmodium* infected host, reshuffles immune responses temporally, effectively locking the subject in a pro-inflammatory loop. We used mice with a BALB/c background that display a T_H_2 weighted immune response profile and are not otherwise susceptible to *P. berghei* induced cerebral malaria. This work reinforces the importance of APC function in order to mount an effective immune response of the old world RF strain bacteria (*B. duttonii*). This is contradictory to the results from work done on new world RF *B. turicatae* and *B. hermsii* species [29], [30]. Dysfunctional activation of APCs clearly affects the clearance of the bacteria. Several conserved molecular structures of *Plasmodium* have been proposed to act as pathogen-associated molecular patterns (PAMPs) and are activating Toll-like receptors (TLRs) on macrophages and dendritic cells (DCs) [57], such as glycosylphosphatidylinositol (GPI), which is a TLR2 ligand [58]. If immune responses to the RF species *B. duttonii* would depend solely on T-cell independent B-cell activation through TLR2 stimulation one would rather expect a more vigorous response against RF in a co-infection situation with *Plasmodium*. We have shown that induction of ECM during co-infection is a multifactorial event where likely several cell-to-cell signaling mechanisms are deranged.

The present study argues for that ECM as a model for cerebral malaria in human *P. falciparum* infection is a fluid concept. We show that a bacterial infection, in addition to NK65 *P. berghei* malaria, alters the otherwise slow disease progression in non-ECM susceptible mice into an acute ECM-like state. It also underscores the importance of a precisely timed sequence of immunological responses to combat each type of infection. This has implications as how cerebral malaria is perceived and could possibly have implications for other severe malaria syndromes such as severe anemia and respiratory distress syndrome.

## Supporting Information

Figure S1 **Bacteria and parasite numbers in blood.** (A) Numbers of spirochaetes in the blood of mice infected with *B. duttonii* bacteria only, or co-infected with both *P. berghei* and *B. duttonii*. (B) Percentage of erythrocytes containing malaria parasites (designated parasitemia) in mice infected with *P. berghei*, or co-infected with both *P. berghei* and *B. duttonii*. (▪) indicates mice infected with both *P. berghei* and *B. duttonii*, (○) indicates animals infected with *B. duttonii* and (Δ) mice infected with *P. berghei*.
(PDF)

Figure S2 **Gating strategy of dendritic cells.** Spleen mononuclear cells (SMC) were extracted on consecutive days from single and co-infected mice. Splenocytes were stained and acquired by flow cytometry. Scatter plots indicates the relative fluorescence intensity of CD11c+ cells with PE-labeled CD83 on the y axis and APC-labeled MHCII on the x-axis. Representative examples from all time points and types of infection are displayed.
(PDF)

Figure S3 **Gating strategy of MΦs.** Spleen mononuclear cells (SMC) were extracted on consecutive days from single and co-infected mice. Splenocytes were stained and acquired by flow cytometry. Scatter plots indicates the relative fluorescence intensity of F4/80+ cells with PE-labeled CD86 on the y-axis and APC-labeled MHCII on the x-axis. Representative examples from all time points and types of infection are displayed.
(PDF)

Figure S4 **Mean fluorescence intensity (MFI) of CD83 on dendritic cells.** (A) The MFI) of PE labeled CD83 in CD11c+ DCs. (B) The MFI of PE labeled CD83 in CD11c+MHCII+^low^ cells. (C) The MFI of PE labeled CD83 in CD11c+MHCII+^high^ cells, (▪) indicates mice infected with both *P. berghei* and *B. duttonii*, (○) indicates animals infected with *B. duttonii* and (Δ) mice infected with *P. berghei*.
(PDF)

Figure S5 **Mean fluorescence intensity (MFI) of CD86 on MΦs.** (A) The MFI of PE labeled CD86 in F4/80+ cells. (B) The MFI of PE labeled CD86 in F4/80+/MHCII+^low^ cells. (C) The MFI of PE labeled CD86 in F4/80+/MHCII+^high^ cells, (▪) indicates mice infected with both *P. berghei* and *B. duttonii*, (○) indicates animals infected with *B. duttonii* and (Δ) mice infected with *P. berghei*.
(PDF)

Table S1 **Significant (P<0.05, Wilcoxon rank test) differences between time points in** **Figures 3** **–** **5** **are displayed in bold while non-significant values are displayed normally.**
(PDF)

Table S2 **Significant (P<0.05, Wilcoxon rank test) differences between time points in** **Figures 3** **–** **5** **are displayed in bold while non-significant values are displayed normally.**
(PDF)

Table S3 **Significant (P<0.05, Wilcoxon rank test) differences between time points in** **Figures 3** **–** **5** **are displayed in bold while non-significant values are displayed normally.**
(PDF)
