# Supplementary figures and images for: Maladjusted Host Immune Responses Induce Experimental Cerebral Malaria-Like Pathology in a Murine Borrelia and Plasmodium Co-Infection Model
Source: PLoS One. 2014 Jul 30;9(7):e103295. doi: 10.1371/journal.pone.0103295 (PMC4116174; doi:10.1371/journal.pone.0103295)

**A**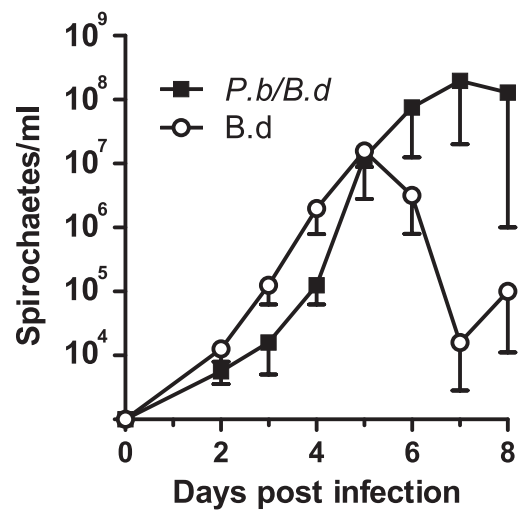**B**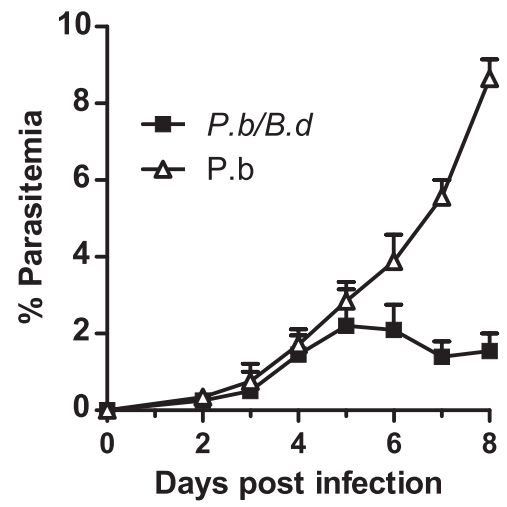

Supplement: Figure S1 — Bacteria and parasite numbers in blood. (A) Numbers of spirochaetes in the blood of mice infected with B. duttonii bacteria only, or co-infected with both P. berghei and B. duttonii. (B) Percentage of erythrocytes containing malaria parasites (designated parasitemia) in mice infected with P. berghei, or co-infected with both P. berghei and B. duttonii. (▪) indicates mice infected with both P. berghei and B. duttonii, (○) indicates animals infected with B. duttonii and (Δ) mice infected with P. berghei. (PDF) [file pone.0103295.s001.pdf]

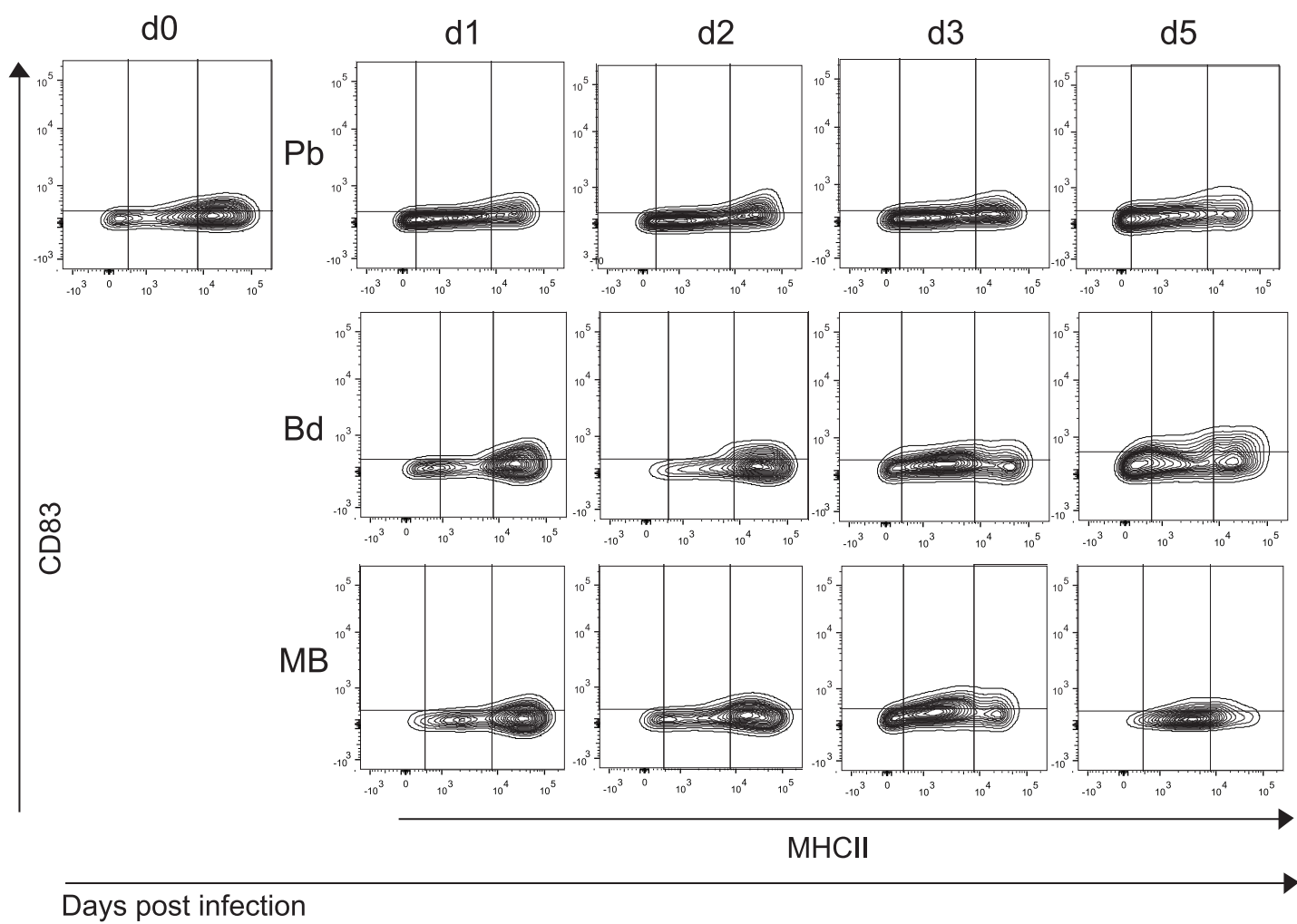

Supplement: Figure S2 — Gating strategy of dendritic cells. Spleen mononuclear cells (SMC) were extracted on consecutive days from single and co-infected mice. Splenocytes were stained and acquired by flow cytometry. Scatter plots indicates the relative fluorescence intensity of CD11c+ cells with PE-labeled CD83 on the y axis and APC-labeled MHCII on the x-axis. Representative examples from all time points and types of infection are displayed. (PDF) [file pone.0103295.s002.pdf]

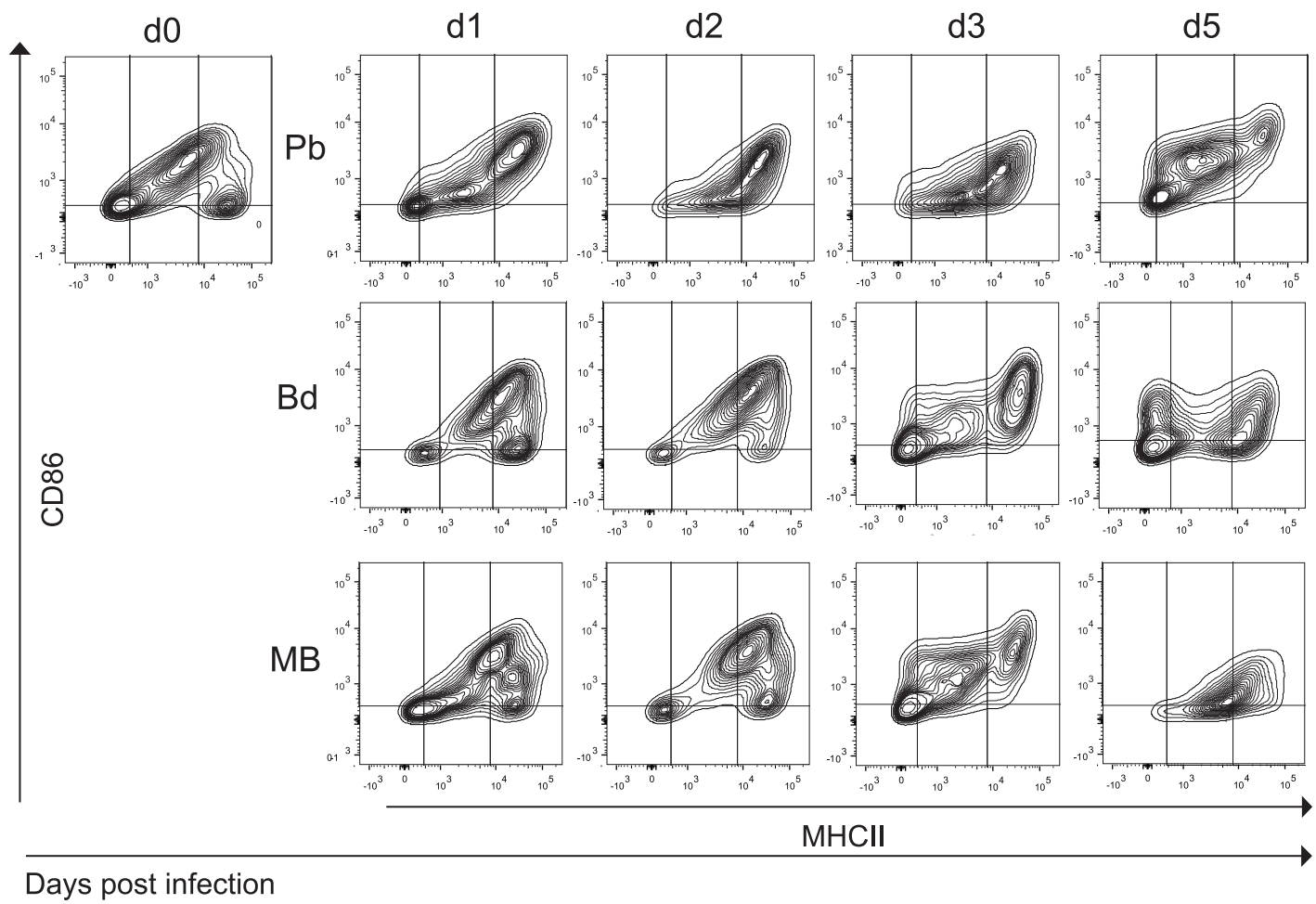

Supplement: Figure S3 — Gating strategy of MΦs. Spleen mononuclear cells (SMC) were extracted on consecutive days from single and co-infected mice. Splenocytes were stained and acquired by flow cytometry. Scatter plots indicates the relative fluorescence intensity of F4/80+ cells with PE-labeled CD86 on the y-axis and APC-labeled MHCII on the x-axis. Representative examples from all time points and types of infection are displayed. (PDF) [file pone.0103295.s003.pdf]

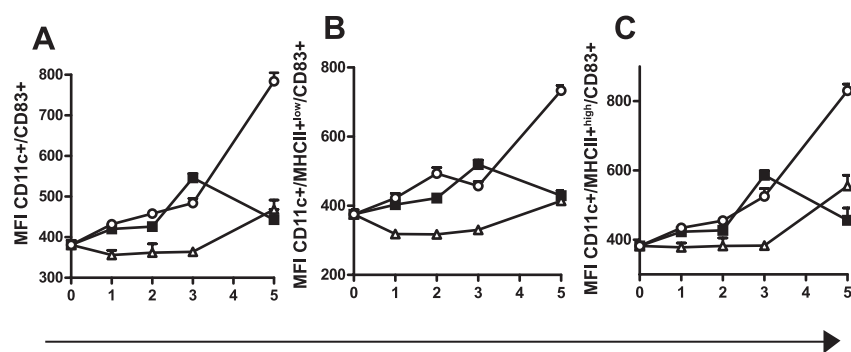

Days post infection

- *P.b/B.d*
- ▲ *P.b*
- *B.d*

Supplement: Figure S4 — Mean fluorescence intensity (MFI) of CD83 on dendritic cells. (A) The MFI) of PE labeled CD83 in CD11c+ DCs. (B) The MFI of PE labeled CD83 in CD11c+MHCII+low cells. (C) The MFI of PE labeled CD83 in CD11c+MHCII+high cells, (▪) indicates mice infected with both P. berghei and B. duttonii, (○) indicates animals infected with B. duttonii and (Δ) mice infected with P. berghei. (PDF) [file pone.0103295.s004.pdf]

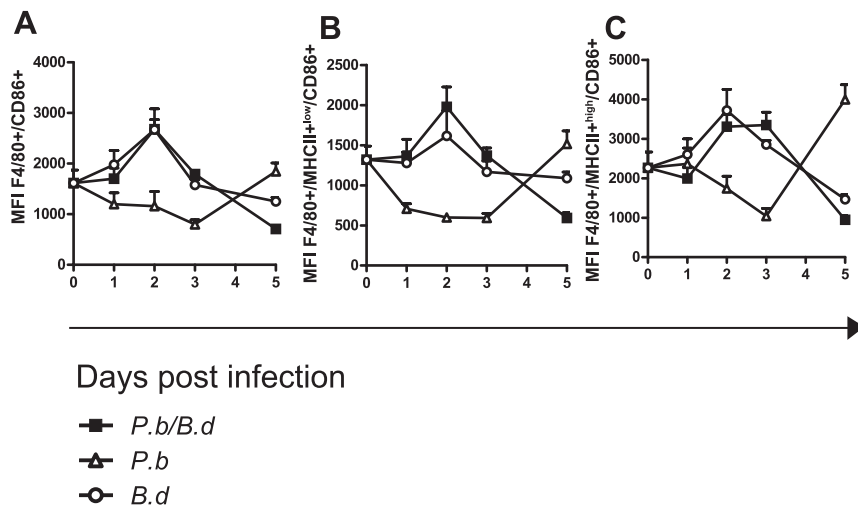

Supplement: Figure S5 — Mean fluorescence intensity (MFI) of CD86 on MΦs. (A) The MFI of PE labeled CD86 in F4/80+ cells. (B) The MFI of PE labeled CD86 in F4/80+/MHCII+low cells. (C) The MFI of PE labeled CD86 in F4/80+/MHCII+high cells, (▪) indicates mice infected with both P. berghei and B. duttonii, (○) indicates animals infected with B. duttonii and (Δ) mice infected with P. berghei. (PDF) [file pone.0103295.s005.pdf]
