# Supplementary material for: Maladjusted Host Immune Responses Induce Experimental Cerebral Malaria-Like Pathology in a Murine Borrelia and Plasmodium Co-Infection Model
Source: PLoS One. 2014 Jul 30;9(7):e103295. doi: 10.1371/journal.pone.0103295 (PMC4116174; doi:10.1371/journal.pone.0103295)
Supplement: Table S1 — Significant (P<0.05, Wilcoxon rank test) differences between time points in Figures 3 – 5 are displayed in bold while non-significant values are displayed normally. (PDF) [file pone.0103295.s006.pdf]

**Table S1. Wilcoxon rank test P-value comparisons in Figure 3**

**IL-10**

| Timepoint | Cd0 | Bd1 | Bd3 | Bd5 | Bd8 | Md1 | Md3 | Md5 | Md8 | MBd1 | MBd3 | MBd5 | MBd8 |
|---|---|---|---|---|---|---|---|---|---|---|---|---|---|
| **Cd0** | 1 | 0.57143 | **0.00433** | 0.24675 | 0.93074 | 1 | **0.0303** | **0.00866** | 0.84127 | **0.00433** | 0.79221 | 0.93074 | **0.00433** |
| **Bd1** | 0.57143 | 1 | **0.02381** | 1 | 1 | 0.38095 | 0.16667 | **0.02381** | 0.25 | **0.02381** | 0.71429 | 0.54762 | **0.02381** |
| **Bd3** | **0.00433** | **0.02381** | 1 | **0.00216** | **0.01515** | **0.00216** | **0.00216** | **0.00216** | **0.00433** | **0.00216** | **0.00216** | **0.00216** | 1 |
| **Bd5** | 0.24675 | 1 | **0.00216** | 1 | 0.81818 | 0.30952 | 0.39394 | **0.00216** | 0.12554 | **0.00216** | 0.58874 | 0.30952 | **0.00866** |
| **Bd8** | 0.93074 | 1 | **0.01515** | 0.81818 | 1 | 0.81818 | 0.48485 | 0.39394 | 0.79221 | 0.06494 | 1 | 0.69913 | **0.02597** |
| **Md1** | 1 | 0.38095 | **0.00216** | 0.30952 | 0.81818 | 1 | 0.09307 | **0.01515** | 0.93074 | **0.00216** | 0.93723 | 0.69913 | **0.00433** |
| **Md3** | **0.0303** | 0.16667 | **0.00216** | 0.39394 | 0.48485 | 0.09307 | 1 | **0.00216** | **0.0303** | **0.00216** | 0.17965 | 0.30952 | **0.01515** |
| **Md5** | **0.00866** | **0.02381** | **0.00216** | **0.00216** | 0.39394 | **0.01515** | **0.00216** | 1 | **0.0303** | **0.00216** | 0.06494 | 0.39394 | **0.00216** |
| **Md8** | 0.84127 | 0.25 | **0.00433** | 0.12554 | 0.79221 | 0.93074 | **0.0303** | **0.0303** | 1 | **0.00433** | 0.66234 | 0.93074 | **0.00433** |
| **MBd1** | **0.00433** | **0.02381** | **0.00216** | **0.00216** | 0.06494 | **0.00216** | **0.00216** | **0.00216** | **0.00433** | 1 | **0.00216** | **0.00639** | 0.2615 |
| **MBd3** | 0.79221 | 0.71429 | **0.00216** | 0.58874 | 1 | 0.93723 | 0.17965 | 0.06494 | 0.66234 | **0.00216** | 1 | 0.74835 | **0.00433** |
| **MBd5** | 0.93074 | 0.54762 | **0.00216** | 0.30952 | 0.69913 | 0.69913 | 0.30952 | 0.39394 | 0.93074 | **0.00639** | 0.74835 | 1 | **0.00866** |
| **MBd8** | **0.00433** | **0.02381** | 1 | **0.00866** | **0.02597** | **0.00433** | **0.01515** | **0.00216** | **0.00433** | 0.2615 | **0.00433** | **0.00866** | 1 |

**IL-1beta**

| Timepoint | Cd0 | Bd1 | Bd3 | Bd5 | Bd8 | Md1 | Md3 | Md5 | Md8 | MBd1 | MBd3 | MBd5 | MBd8 |
|---|---|---|---|---|---|---|---|---|---|---|---|---|---|
| **Cd0** | 1 | 0.57143 | 1 | **0.0303** | 0.79221 | 1 | 0.17749 | 0.08225 | 0.05556 | **0.00433** | 0.79221 | 0.84127 | **0.00794** |
| **Bd1** | 0.57143 | 1 | 0.16667 | **0.02381** | 0.2619 | 0.38095 | **0.02381** | **0.02381** | **0.03571** | **0.02381** | 0.71429 | 0.78571 | **0.03571** |
| **Bd3** | 1 | 0.16667 | 1 | **0.00216** | 0.30952 | 0.69913 | **0.00216** | **0.00216** | **0.00433** | **0.00216** | 0.69913 | 0.66234 | **0.00433** |
| **Bd5** | **0.0303** | **0.02381** | **0.00216** | 1 | **0.02597** | 0.09307 | 0.06494 | 0.48485 | 0.66234 | **0.00216** | 0.09307 | 0.12554 | **0.00433** |
| **Bd8** | 0.79221 | 0.2619 | 0.30952 | **0.02597** | 1 | 0.48485 | 0.30952 | **0.02597** | **0.0303** | **0.00216** | 0.58874 | 0.5368 | **0.00866** |
| **Md1** | 1 | 0.38095 | 0.69913 | 0.09307 | 0.48485 | 1 | 0.09307 | 0.06494 | 0.05195 | **0.00216** | 0.93723 | 1 | **0.00866** |
| **Md3** | 0.17749 | **0.02381** | **0.00216** | 0.06494 | 0.30952 | 0.09307 | 1 | 0.17965 | 0.12554 | **0.00216** | 0.09307 | 0.12554 | **0.00433** |
| **Md5** | 0.08225 | **0.02381** | **0.00216** | 0.48485 | **0.02597** | 0.06494 | 0.17965 | 1 | 0.66234 | **0.00216** | 0.06494 | 0.08225 | **0.00433** |
| **Md8** | 0.05556 | **0.03571** | **0.00433** | 0.66234 | **0.0303** | 0.05195 | 0.12554 | 0.66234 | 1 | **0.00433** | 0.05195 | 0.09524 | **0.00794** |
| **MBd1** | **0.00433** | **0.02381** | **0.00216** | **0.00216** | **0.00216** | **0.00216** | **0.00216** | **0.00216** | **0.00433** | 1 | **0.00216** | **0.01041** | 0.46419 |
| **MBd3** | 0.79221 | 0.71429 | 0.69913 | 0.09307 | 0.58874 | 0.93723 | 0.09307 | 0.06494 | 0.05195 | **0.00216** | 1 | 1 | **0.00866** |
| **MBd5** | 0.84127 | 0.78571 | 0.66234 | 0.12554 | 0.5368 | 1 | 0.12554 | 0.08225 | 0.09524 | **0.01041** | 1 | 1 | **0.03175** |
| **MBd8** | **0.00794** | **0.03571** | **0.00433** | **0.00433** | **0.00866** | **0.00866** | **0.00433** | **0.00433** | **0.00794** | 0.46419 | **0.00866** | **0.03175** | 1 |

**TNF-alpha**

| Timepoint | Cd0 | Bd1 | Bd3 | Bd5 | Bd8 | Md1 | Md3 | Md5 | Md8 | MBd1 | MBd3 | MBd5 | MBd8 |
|---|---|---|---|---|---|---|---|---|---|---|---|---|---|
| **Cd0** | 1 | **0.03571** | **0.00866** | 0.42857 | **0.00433** | **0.0303** | 0.5368 | 0.42857 | **0.00794** | **0.00433** | 0.24675 | **0.00433** | **0.00794** |
| **Bd1** | **0.03571** | 1 | 0.09524 | **0.02381** | 0.09524 | 1 | **0.02381** | **0.04762** | **0.03571** | **0.02381** | 0.2619 | **0.04762** | **0.03571** |
| **Bd3** | **0.00866** | 0.09524 | 1 | **0.00216** | **0.00216** | 0.30952 | **0.04113** | **0.02597** | **0.00433** | **0.00216** | 1 | **0.00216** | **0.00433** |
| **Bd5** | 0.42857 | **0.02381** | **0.00216** | 1 | **0.00216** | **0.00433** | 0.09307 | 0.69913 | **0.00433** | **0.00216** | 0.09307 | **0.00216** | **0.00433** |
| **Bd8** | **0.00433** | 0.09524 | **0.00216** | **0.00216** | 1 | 0.13203 | **0.00216** | **0.00216** | 0.66234 | 0.58874 | **0.01515** | 0.69913 | **0.01732** |
| **Md1** | **0.0303** | 1 | 0.30952 | **0.00433** | 0.13203 | 1 | **0.04113** | **0.01515** | **0.00433** | **0.00216** | 0.30952 | **0.01515** | **0.00433** |
| **Md3** | 0.5368 | **0.02381** | **0.04113** | 0.09307 | **0.00216** | **0.04113** | 1 | 0.24026 | **0.00433** | **0.00216** | 0.39394 | **0.00216** | **0.00433** |
| **Md5** | 0.42857 | **0.04762** | **0.02597** | 0.69913 | **0.00216** | **0.01515** | 0.24026 | 1 | **0.00433** | **0.00216** | 0.13203 | **0.00216** | **0.00433** |
| **Md8** | **0.00794** | **0.03571** | **0.00433** | **0.00433** | 0.66234 | **0.00433** | **0.00433** | **0.00433** | 1 | 0.66234 | **0.00433** | 0.79221 | **0.01587** |
| **MBd1** | **0.00433** | **0.02381** | **0.00216** | **0.00216** | 0.58874 | **0.00216** | **0.00216** | **0.00216** | 0.66234 | 1 | **0.00216** | 1 | **0.00866** |
| **MBd3** | 0.24675 | 0.2619 | 1 | 0.09307 | **0.01515** | 0.30952 | 0.39394 | 0.13203 | **0.00433** | **0.00216** | 1 | **0.00433** | **0.00433** |
| **MBd5** | **0.00433** | **0.04762** | **0.00216** | **0.00216** | 0.69913 | **0.01515** | **0.00216** | **0.00216** | 0.79221 | 1 | **0.00433** | 1 | **0.00866** |
| **MBd8** | **0.00794** | **0.03571** | **0.00433** | **0.00433** | **0.01732** | **0.00433** | **0.00433** | **0.00433** | **0.01587** | **0.00866** | **0.00433** | **0.00866** | 1 |

**CD4+/CD25+$^{high}$/FoxP3+**

| Timepoint | Cd0 | Bd1 | Bd2 | Bd3 | Bd5 | Md1 | Md2 | Md3 | Md5 | MBd1 | MBd2 | MBd3 | MBd5 |
|---|---|---|---|---|---|---|---|---|---|---|---|---|---|
| **Cd0** | 1 | **0.00866** | **0.00216** | **0.00216** | **0.00216** | **0.00216** | **0.00216** | **0.005** | **0.00216** | 0.39394 | 0.24026 | **0.00216** | **0.00216** |
| **Bd1** | **0.00866** | 1 | **0.00216** | **0.00216** | **0.00216** | **0.00866** | **0.00433** | 0.17273 | **0.00216** | **0.02597** | **0.00216** | 0.09307 | **0.04113** |
| **Bd2** | **0.00216** | **0.00216** | 1 | **0.00216** | **0.00216** | **0.00216** | **0.00216** | **0.005** | **0.00216** | **0.04113** | 0.09307 | **0.00216** | **0.00216** |
| **Bd3** | **0.00216** | **0.00216** | **0.00216** | 1 | **0.00433** | **0.00216** | **0.00216** | **0.005** | **0.00216** | **0.00216** | **0.00216** | **0.00216** | **0.00216** |
| **Bd5** | **0.00216** | **0.00216** | **0.00216** | **0.00433** | 1 | **0.00216** | **0.00216** | **0.005** | **0.00216** | **0.00216** | **0.00216** | **0.00216** | **0.00216** |
| **Md1** | **0.00216** | **0.00866** | **0.00216** | **0.00216** | **0.00216** | 1 | 0.39394 | **0.01291** | **0.00866** | **0.00216** | **0.00216** | 0.06494 | 0.39394 |
| **Md2** | **0.00216** | **0.00433** | **0.00216** | **0.00216** | **0.00216** | 0.39394 | 1 | **0.005** | 0.30952 | **0.00216** | **0.00216** | **0.01515** | 0.13203 |
| **Md3** | **0.005** | 0.17273 | **0.005** | **0.005** | **0.005** | **0.01291** | **0.005** | 1 | **0.005** | **0.01291** | **0.005** | 0.93607 | 0.22895 |
| **Md5** | **0.00216** | **0.00216** | **0.00216** | **0.00216** | **0.00216** | **0.00866** | 0.30952 | **0.005** | 1 | **0.00216** | **0.00216** | **0.00433** | 0.06494 |
| **MBd1** | 0.39394 | **0.02597** | **0.04113** | **0.00216** | **0.00216** | **0.00216** | **0.00216** | **0.01291** | **0.00216** | 1 | 1 | **0.00866** | **0.00216** |
| **MBd2** | 0.24026 | **0.00216** | 0.09307 | **0.00216** | **0.00216** | **0.00216** | **0.00216** | **0.005** | **0.00216** | 1 | 1 | **0.00216** | **0.00216** |
| **MBd3** | **0.00216** | 0.09307 | **0.00216** | **0.00216** | **0.00216** | 0.06494 | **0.01515** | 0.93607 | **0.00433** | **0.00866** | **0.00216** | 1 | 0.30952 |
| **MBd5** | **0.00216** | **0.04113** | **0.00216** | **0.00216** | **0.00216** | 0.39394 | 0.13203 | 0.22895 | 0.06494 | **0.00216** | **0.00216** | 0.30952 | 1 |
