# Supplementary material for: Maladjusted Host Immune Responses Induce Experimental Cerebral Malaria-Like Pathology in a Murine Borrelia and Plasmodium Co-Infection Model
Source: PLoS One. 2014 Jul 30;9(7):e103295. doi: 10.1371/journal.pone.0103295 (PMC4116174; doi:10.1371/journal.pone.0103295)
Supplement: Table S2 — Significant (P<0.05, Wilcoxon rank test) differences between time points in Figures 3 – 5 are displayed in bold while non-significant values are displayed normally. (PDF) [file pone.0103295.s007.pdf]

Table S2. Wilcoxon rank test P-value comparisons in Figure 4

Fq CD11c+/MHCII+

Timepoint	Cd0	Bd1	Bd2	Bd3	Bd5	Md1	Md2	Md3	Md5	MBd1	MBd2	MBd3	MBd5
**Cd0**	1	0.24026	**0.005**	0.69913	**0.00216**	0.33241	0.58874	0.69913	0.09307	0.06508	**0.00433**	1	0.09213
**Bd1**	0.24026	1	**0.005**	0.68788	**0.00216**	0.5745	1	0.13203	0.09307	**0.01291**	**0.00216**	0.1994	0.06508
**Bd2**	**0.005**	**0.005**	1	**0.005**	**0.005**	**0.00492**	**0.005**	**0.005**	**0.005**	**0.01981**	**0.00813**	**0.005**	0.3768
**Bd3**	0.69913	0.68788	**0.005**	1	**0.00216**	1	0.80953	0.63036	**0.04113**	**0.03035**	**0.02597**	0.58874	**0.04495**
**Bd5**	**0.00216**	**0.00216**	**0.005**	**0.00216**	1	**0.005**	**0.00216**	**0.00216**	**0.00433**	**0.005**	**0.00216**	**0.00216**	**0.005**
**Md1**	0.33241	0.5745	**0.00492**	1	**0.005**	1	0.5745	0.17273	**0.04495**	**0.00492**	**0.005**	0.22731	**0.04458**
**Md2**	0.58874	1	**0.005**	0.80953	**0.00216**	0.5745	1	0.48485	**0.02597**	**0.03035**	**0.01515**	0.58874	**0.03671**
**Md3**	0.69913	0.13203	**0.005**	0.63036	**0.00216**	0.17273	0.48485	1	**0.01515**	0.09213	**0.03006**	0.81818	0.09213
**Md5**	0.09307	0.09307	**0.005**	**0.04113**	**0.00433**	**0.04495**	**0.02597**	**0.01515**	1	**0.005**	**0.00216**	**0.00866**	**0.00813**
**MBd1**	0.06508	**0.01291**	**0.01981**	**0.03035**	**0.005**	**0.00492**	**0.03035**	0.09213	**0.005**	1	0.29711	**0.03035**	0.57383
**MBd2**	**0.00433**	**0.00216**	**0.00813**	**0.02597**	**0.00216**	**0.005**	**0.01515**	**0.03006**	**0.00216**	0.29711	1	**0.00216**	0.5745
**MBd3**	1	0.1994	**0.005**	0.58874	**0.00216**	0.22731	0.58874	0.81818	**0.00866**	**0.03035**	**0.00216**	1	0.09213
**MBd5**	0.09213	0.06508	0.3768	**0.04495**	**0.005**	**0.04458**	**0.03671**	0.09213	**0.00813**	0.57383	0.5745	0.09213	1

Fq CD11c+/MHCII+/MHCII+^{high}

Timepoint	Cd0	Bd1	Bd2	Bd3	Bd5	Md1	Md2	Md3	Md5	MBd1	MBd2	MBd3	MBd5
**Cd0**	1	**0.00216**	**0.00216**	**0.00216**	**0.00216**	**0.00216**	**0.00216**	**0.00216**	**0.00216**	1	0.06494	**0.00216**	**0.00216**
**Bd1**	**0.00216**	1	0.42253	**0.00216**	**0.00216**	**0.00216**	**0.00216**	**0.00216**	**0.00216**	**0.00433**	**0.02597**	**0.00216**	**0.00216**
**Bd2**	**0.00216**	0.42253	1	**0.00216**	**0.00216**	**0.00216**	**0.00216**	**0.00216**	**0.00216**	**0.00433**	**0.00216**	**0.00216**	**0.00216**
**Bd3**	**0.00216**	**0.00216**	**0.00216**	1	**0.00216**	0.13203	0.93723	0.30952	**0.00216**	**0.00216**	**0.00216**	0.69913	0.69913
**Bd5**	**0.00216**	**0.00216**	**0.00216**	**0.00216**	1	**0.00216**	**0.00216**	**0.00216**	**0.00216**	**0.00216**	**0.00216**	**0.00216**	**0.01515**
**Md1**	**0.00216**	**0.00216**	**0.00216**	0.13203	**0.00216**	1	0.17965	**0.04113**	**0.01515**	**0.00216**	**0.00216**	0.48485	0.81818
**Md2**	**0.00216**	**0.00216**	**0.00216**	0.93723	**0.00216**	0.17965	1	0.39394	**0.00866**	**0.00216**	**0.00216**	0.81818	1
**Md3**	**0.00216**	**0.00216**	**0.00216**	0.30952	**0.00216**	**0.04113**	0.39394	1	**0.00216**	**0.00216**	**0.00216**	0.24026	0.48485
**Md5**	**0.00216**	**0.00216**	**0.00216**	**0.00216**	**0.00216**	**0.01515**	**0.00866**	**0.00216**	1	**0.00216**	**0.00216**	**0.01515**	0.17965
**MBd1**	1	**0.00433**	**0.00433**	**0.00216**	**0.00216**	**0.00216**	**0.00216**	**0.00216**	**0.00216**	1	0.39394	**0.00216**	**0.00216**
**MBd2**	0.06494	**0.02597**	**0.00216**	**0.00216**	**0.00216**	**0.00216**	**0.00216**	**0.00216**	**0.00216**	0.39394	1	**0.00216**	**0.00216**
**MBd3**	**0.00216**	**0.00216**	**0.00216**	0.69913	**0.00216**	0.48485	0.81818	0.24026	**0.01515**	**0.00216**	**0.00216**	1	0.93723
**MBd5**	**0.00216**	**0.00216**	**0.00216**	0.69913	**0.01515**	0.81818	1	0.48485	0.17965	**0.00216**	**0.00216**	0.93723	1

Fq CD11c+/CD83+

Timepoint	Cd0	Bd1	Bd2	Bd3	Bd5	Md1	Md2	Md3	Md5	MBd1	MBd2	MBd3	MBd5
**Cd0**	1	0.13203	1	**0.01515**	**0.00433**	**0.00433**	**0.00866**	**0.01515**	0.87256	**0.00216**	**0.02447**	0.06494	0.06508
**Bd1**	0.13203	1	0.69913	0.24026	**0.04113**	**0.00216**	**0.00216**	**0.00216**	0.24026	**0.00216**	**0.005**	0.58874	**0.005**
**Bd2**	1	0.69913	1	**0.02597**	**0.02597**	**0.01515**	**0.04113**	**0.04113**	0.81818	**0.01027**	0.09213	0.58874	0.06508
**Bd3**	**0.01515**	0.24026	**0.02597**	1	0.81818	**0.00216**	**0.00216**	**0.00216**	**0.01515**	**0.00216**	**0.005**	0.24026	**0.005**
**Bd5**	**0.00433**	**0.04113**	**0.02597**	0.81818	1	**0.00216**	**0.00216**	**0.00216**	**0.01027**	**0.00216**	**0.005**	0.10869	**0.005**
**Md1**	**0.00433**	**0.00216**	**0.01515**	**0.00216**	**0.00216**	1	0.48485	0.30952	**0.00866**	0.69913	0.12754	**0.00216**	0.22895
**Md2**	**0.00866**	**0.00216**	**0.04113**	**0.00216**	**0.00216**	0.48485	1	0.74835	**0.01515**	0.10869	0.17273	**0.00216**	0.80985
**Md3**	**0.01515**	**0.00216**	**0.04113**	**0.00216**	**0.00216**	0.30952	0.74835	1	**0.02472**	**0.02597**	0.22895	**0.00216**	0.93607
**Md5**	0.87256	0.24026	0.81818	**0.01515**	**0.01027**	**0.00866**	**0.01515**	**0.02472**	1	**0.00433**	0.06508	0.12687	0.06508
**MBd1**	**0.00216**	**0.00216**	**0.01027**	**0.00216**	**0.00216**	0.69913	0.10869	**0.02597**	**0.00433**	1	**0.02002**	**0.00216**	**0.03035**
**MBd2**	**0.02447**	**0.005**	0.09213	**0.005**	**0.005**	0.12754	0.17273	0.22895	0.06508	**0.02002**	1	**0.00813**	0.42089
**MBd3**	0.06494	0.58874	0.58874	0.24026	0.10869	**0.00216**	**0.00216**	**0.00216**	0.12687	**0.00216**	**0.00813**	1	**0.00813**
**MBd5**	0.06508	**0.005**	0.06508	**0.005**	**0.005**	0.22895	0.80985	0.93607	0.06508	**0.03035**	0.42089	**0.00813**	1

Fq CD11c+/MHCII+^{low}/CD83+

Timepoint	Cd0	Bd1	Bd2	Bd3	Bd5	Md1	Md2	Md3	Md5	MBd1	MBd2	MBd3	MBd5
**Cd0**	1	0.48485	0.48485	**0.02597**	**0.00216**	**0.00492**	**0.00216**	**0.00216**	**0.005**	**0.00216**	**0.04113**	**0.00866**	0.13203
**Bd1**	0.48485	1	0.93723	**0.01515**	**0.00216**	**0.01981**	**0.01027**	0.10869	**0.005**	**0.00433**	0.33582	0.09307	0.58874
**Bd2**	0.48485	0.93723	1	**0.01515**	**0.00216**	**0.00492**	**0.00216**	**0.00866**	**0.005**	**0.00216**	0.09307	**0.00866**	0.24026
**Bd3**	**0.02597**	**0.01515**	**0.01515**	1	0.63036	**0.00492**	**0.00216**	**0.00216**	**0.02002**	**0.00216**	**0.00433**	0.30952	**0.00433**
**Bd5**	**0.00216**	**0.00216**	**0.00216**	0.63036	1	**0.00492**	**0.00216**	**0.00216**	**0.02002**	**0.00216**	**0.00216**	0.06494	**0.00216**
**Md1**	**0.00492**	**0.01981**	**0.00492**	**0.00492**	**0.00492**	1	0.57383	**0.04458**	**0.00485**	0.12687	0.12687	**0.00492**	**0.01575**
**Md2**	**0.00216**	**0.01027**	**0.00216**	**0.00216**	**0.00216**	0.57383	1	**0.02472**	**0.005**	0.14883	**0.02472**	**0.00216**	**0.00433**
**Md3**	**0.00216**	0.10869	**0.00866**	**0.00216**	**0.00216**	**0.04458**	**0.02472**	1	**0.005**	**0.00866**	0.58874	**0.00216**	0.24026
**Md5**	**0.005**	**0.005**	**0.005**	**0.02002**	**0.02002**	**0.00485**	**0.005**	**0.005**	1	**0.005**	**0.005**	**0.02002**	**0.005**
**MBd1**	**0.00216**	**0.00433**	**0.00216**	**0.00216**	**0.00216**	0.12687	0.14883	**0.00866**	**0.005**	1	**0.01515**	**0.00216**	**0.00433**
**MBd2**	**0.04113**	0.33582	0.09307	**0.00433**	**0.00216**	0.12687	**0.02472**	0.58874	**0.005**	**0.01515**	1	**0.00216**	0.58874
**MBd3**	**0.00866**	0.09307	**0.00866**	0.30952	0.06494	**0.00492**	**0.00216**	**0.00216**	**0.02002**	**0.00216**	**0.00216**	1	**0.00866**
**MBd5**	0.13203	0.58874	0.24026	**0.00433**	**0.00216**	**0.01575**	**0.00433**	0.24026	**0.005**	**0.00433**	0.58874	**0.00866**	1

Fq CD11c+/MHCII+^{high}/CD83+

Timepoint	Cd0	Bd1	Bd2	Bd3	Bd5	Md1	Md2	Md3	Md5	MBd1	MBd2	MBd3	MBd5
**Cd0**	1	0.07765	0.6884	**0.005**	**0.00216**	0.13203	0.06494	0.24026	**0.00216**	**0.00216**	**0.00216**	**0.005**	0.17273
**Bd1**	0.07765	1	0.22895	**0.01291**	**0.00216**	0.81818	0.39394	0.58874	**0.00216**	**0.00216**	**0.00216**	**0.01014**	0.06508
**Bd2**	0.6884	0.22895	1	**0.00492**	**0.005**	0.22895	**0.03035**	0.37764	**0.005**	**0.02002**	0.29711	**0.00492**	1
**Bd3**	**0.005**	**0.01291**	**0.00492**	1	**0.02002**	0.09213	0.37764	**0.03035**	**0.005**	**0.005**	**0.005**	0.46961	**0.01981**
**Bd5**	**0.00216**	**0.00216**	**0.005**	**0.02002**	1	**0.00433**	**0.00866**	**0.00216**	**0.00216**	**0.00216**	**0.00216**	**0.00813**	**0.005**
**Md1**	0.13203	0.81818	0.22895	0.09213	**0.00433**	1	0.39394	0.58874	**0.00216**	**0.00433**	**0.01515**	0.22895	0.17273
**Md2**	0.06494	0.39394	**0.03035**	0.37764	**0.00866**	0.39394	1	0.39394	**0.00216**	**0.00216**	**0.00216**	0.37764	0.09213
**Md3**	0.24026	0.58874	0.37764	**0.03035**	**0.00216**	0.58874	0.39394	1	**0.00216**	**0.00216**	**0.00216**	0.06508	0.06508
**Md5**	**0.00216**	**0.00216**	**0.005**	**0.005**	**0.00216**	**0.00216**	**0.00216**	**0.00216**	1	**0.00216**	**0.00216**	**0.005**	**0.005**
**MBd1**	**0.00216**	**0.00216**	**0.02002**	**0.005**	**0.00216**	**0.00433**	**0.00216**	**0.00216**	**0.00216**	1	**0.01515**	**0.005**	**0.01291**
**MBd2**	**0.00216**	**0.00216**	0.29711	**0.005**	**0.00216**	**0.01515**	**0.00216**	**0.00216**	**0.00216**	**0.01515**	1	**0.005**	0.17273
**MBd3**	**0.005**	**0.01014**	**0.00492**	0.46961	**0.00813**	0.22895	0.37764	0.06508	**0.005**	**0.005**	**0.005**	1	**0.04458**
**MBd5**	0.17273	0.06508	1	**0.01981**	**0.005**	0.17273	0.09213	0.06508	**0.005**	**0.01291**	0.17273	**0.04458**	1
