# Supplementary material for: Maladjusted Host Immune Responses Induce Experimental Cerebral Malaria-Like Pathology in a Murine Borrelia and Plasmodium Co-Infection Model
Source: PLoS One. 2014 Jul 30;9(7):e103295. doi: 10.1371/journal.pone.0103295 (PMC4116174; doi:10.1371/journal.pone.0103295)
Supplement: Table S3 — Significant (P<0.05, Wilcoxon rank test) differences between time points in Figures 3 – 5 are displayed in bold while non-significant values are displayed normally. (PDF) [file pone.0103295.s008.pdf]

Table S3. Wilcoxon rank test P-value comparisons in Figure 5

Fq F4/80+/MHCII+

Timepoint	Cd0	Bd1	Bd2	Bd3	Bd5	Md1	Md2	Md3	Md5	MBd1	MBd2	MBd3	MBd5
Cd0	1	0.09307	0.00216	0.00216	0.00216	0.06494	0.005	0.00216	0.00866	0.01515	0.00216	0.00216	0.00216
Bd1	0.09307	1	0.03704	0.01515	0.00216	0.58874	0.03671	0.02472	0.69913	0.30952	0.02597	0.04113	0.00216
Bd2	0.00216	0.03704	1	0.00216	0.00216	0.01612	1	0.63036	0.01515	0.00216	0.81818	0.00216	0.01612
Bd3	0.00216	0.01515	0.00216	1	0.00216	0.00216	0.005	0.00216	0.00216	0.00216	0.00216	0.30952	0.00216
Bd5	0.00216	0.00216	0.00216	0.00216	1	0.00216	0.005	0.00216	0.00216	0.00216	0.00216	0.00216	0.00216
Md1	0.06494	0.58874	0.01612	0.00216	0.00216	1	0.005	0.00216	0.74835	0.48485	0.00216	0.00216	0.00216
Md2	0.005	0.03671	1	0.005	0.005	0.005	1	0.68736	0.005	0.005	1	0.005	0.02447
Md3	0.00216	0.02472	0.63036	0.00216	0.00216	0.00216	0.68736	1	0.00216	0.00216	0.30952	0.00216	0.00216
Md5	0.00866	0.69913	0.01515	0.00216	0.00216	0.74835	0.005	0.00216	1	0.24026	0.00216	0.00216	0.00216
MBd1	0.01515	0.30952	0.00216	0.00216	0.00216	0.48485	0.005	0.00216	0.24026	1	0.00216	0.00216	0.00216
MBd2	0.00216	0.02597	0.81818	0.00216	0.00216	0.00216	1	0.30952	0.00216	0.00216	1	0.00216	0.02597
MBd3	0.00216	0.04113	0.00216	0.30952	0.00216	0.00216	0.005	0.00216	0.00216	0.00216	0.00216	1	0.00216
MBd5	0.00216	0.00216	0.01612	0.00216	0.00216	0.00216	0.02447	0.00216	0.00216	0.00216	0.02597	0.00216	1

Fq F4/80+/MHCII+/MHCII+^{high}

Timepoint	Cd0	Bd1	Bd2	Bd3	Bd5	Md1	Md2	Md3	Md5	MBd1	MBd2	MBd3	MBd5
Cd0	1	0.00216	0.00216	0.04113	0.42253	0.93723	0.02597	0.05424	0.00216	0.00433	0.00216	0.005	0.58874
Bd1	0.00216	1	0.06494	0.00216	0.00216	0.00216	0.02597	0.00216	0.00216	0.09307	0.33582	0.005	0.00216
Bd2	0.00216	0.06494	1	0.00216	0.00216	0.00216	0.24026	0.00866	0.00216	0.93723	0.81818	0.005	0.00216
Bd3	0.04113	0.00216	0.00216	1	0.02597	0.13203	0.00866	0.01515	0.00433	0.00216	0.00216	0.12754	0.69913
Bd5	0.42253	0.00216	0.00216	0.02597	1	0.17965	0.06494	0.1994	0.00216	0.00216	0.00216	0.005	0.14883
Md1	0.93723	0.00216	0.00216	0.13203	0.17965	1	0.00866	0.02597	0.00216	0.00216	0.00216	0.02002	0.81818
Md2	0.02597	0.02597	0.24026	0.00866	0.06494	0.00866	1	0.52111	0.00216	0.24026	0.17965	0.005	0.00866
Md3	0.05424	0.00216	0.00866	0.01515	0.1994	0.02597	0.52111	1	0.00216	0.02597	0.01515	0.005	0.04113
Md5	0.00216	0.00216	0.00216	0.00433	0.00216	0.00216	0.00216	0.00216	1	0.00216	0.00216	0.02002	0.01515
MBd1	0.00433	0.09307	0.93723	0.00216	0.00216	0.00216	0.24026	0.02597	0.00216	1	0.69913	0.005	0.00216
MBd2	0.00216	0.33582	0.81818	0.00216	0.00216	0.00216	0.17965	0.01515	0.00216	0.69913	1	0.005	0.00216
MBd3	0.005	0.005	0.005	0.12754	0.005	0.02002	0.005	0.005	0.02002	0.005	0.005	1	0.37764
MBd5	0.58874	0.00216	0.00216	0.69913	0.14883	0.81818	0.00866	0.04113	0.01515	0.00216	0.00216	0.37764	1

Fq F4/80+/CD86+

Timepoint	Cd0	Bd1	Bd2	Bd3	Bd5	Md1	Md2	Md3	Md5	MBd1	MBd2	MBd3	MBd5
Cd0	1	0.30952	0.00216	0.02597	0.00216	0.74835	0.09307	1	0.00216	0.01027	0.00216	0.04113	0.39394
Bd1	0.30952	1	0.00433	0.13203	0.01515	0.58874	0.81818	0.39394	0.09307	0.06494	0.01515	0.06494	0.58874
Bd2	0.00216	0.00433	1	0.00216	0.00216	0.00216	0.01027	0.00216	0.17965	0.00216	0.48485	0.00216	0.00216
Bd3	0.02597	0.13203	0.00216	1	0.00866	0.01515	0.00866	0.02597	0.00216	0.30952	0.00216	0.06494	0.42253
Bd5	0.00216	0.01515	0.00216	0.00866	1	0.00216	0.00216	0.00216	0.00216	0.00433	0.00216	0.00216	0.00216
Md1	0.74835	0.58874	0.00216	0.01515	0.00216	1	0.1994	0.93723	0.01612	0.00866	0.00216	0.06494	0.39394
Md2	0.09307	0.81818	0.01027	0.00866	0.00216	0.1994	1	0.09307	0.09307	0.00216	0.02597	0.00216	0.13203
Md3	1	0.39394	0.00216	0.02597	0.00216	0.93723	0.09307	1	0.02472	0.00216	0.00433	0.02597	0.39394
Md5	0.00216	0.09307	0.17965	0.00216	0.00216	0.01612	0.09307	0.02472	1	0.00216	0.58874	0.00216	0.06494
MBd1	0.01027	0.06494	0.00216	0.30952	0.00433	0.00866	0.00216	0.00216	0.00216	1	0.00216	0.04113	1
MBd2	0.00216	0.01515	0.48485	0.00216	0.00216	0.00216	0.02597	0.00433	0.58874	0.00216	1	0.00216	0.00866
MBd3	0.04113	0.06494	0.00216	0.06494	0.00216	0.06494	0.00216	0.02597	0.00216	0.04113	0.00216	1	0.39394
MBd5	0.39394	0.58874	0.00216	0.42253	0.00216	0.39394	0.13203	0.39394	0.06494	1	0.00866	0.39394	1

Fq F4/80+/MHCII+^{low}/CD86+

Timepoint	Cd0	Bd1	Bd2	Bd3	Bd5	Md1	Md2	Md3	Md5	MBd1	MBd2	MBd3	MBd5
Cd0	1	0.1994	0.00216	0.02472	0.005	0.00216	0.00216	0.00216	0.005	0.00216	0.03035	0.06494	0.005
Bd1	0.1994	1	0.01515	0.05424	0.06508	0.04113	0.01515	0.00866	0.0063	0.01515	0.37764	0.06494	0.01291
Bd2	0.00216	0.01515	1	0.00216	0.005	0.00216	0.00216	0.00216	0.12754	0.00216	0.37764	0.00216	0.005
Bd3	0.02472	0.05424	0.00216	1	0.6884	0.74835	0.24026	0.17965	0.005	0.01515	0.005	0.06494	0.22895
Bd5	0.005	0.06508	0.005	0.6884	1	0.5745	0.17122	0.12754	0.00492	0.04383	0.00492	0.00813	0.3768
Md1	0.00216	0.04113	0.00216	0.74835	0.5745	1	0.17965	0.09307	0.005	0.04113	0.005	0.07765	0.22895
Md2	0.00216	0.01515	0.00216	0.24026	0.17122	0.17965	1	0.24026	0.005	0.24026	0.005	0.00216	0.47039
Md3	0.00216	0.00866	0.00216	0.17965	0.12754	0.09307	0.24026	1	0.005	0.48485	0.005	0.01515	0.80985
Md5	0.005	0.0063	0.12754	0.005	0.00492	0.005	0.005	0.005	1	0.005	0.06461	0.005	0.00492
MBd1	0.00216	0.01515	0.00216	0.01515	0.04383	0.04113	0.24026	0.48485	0.005	1	0.005	0.00216	0.6884
MBd2	0.03035	0.37764	0.37764	0.005	0.00492	0.005	0.005	0.005	0.06461	0.005	1	0.005	0.00492
MBd3	0.06494	0.06494	0.00216	0.06494	0.00813	0.07765	0.00216	0.01515	0.005	0.00216	0.005	1	0.06508
MBd5	0.005	0.01291	0.005	0.22895	0.3768	0.22895	0.47039	0.80985	0.00492	0.6884	0.00492	0.06508	1

Fq F4/80+/MHCII+^{high}/CD86+

Timepoint	Cd0	Bd1	Bd2	Bd3	Bd5	Md1	Md2	Md3	Md5	MBd1	MBd2	MBd3	MBd5
Cd0	1	0.46883	0.00216	0.00216	0.00216	0.00216	0.00216	0.00866	0.00477	0.17273	0.00216	0.005	0.33582
Bd1	0.46883	1	0.005	0.005	0.005	0.005	0.005	0.03671	0.0047	0.03006	0.005	0.00492	0.52038
Bd2	0.00216	0.005	1	0.87211	0.00216	0.04113	0.74749	0.00866	0.00477	0.005	0.42253	0.62975	0.04113
Bd3	0.00216	0.005	0.87211	1	0.00216	0.00639	0.80953	0.00866	0.00477	0.005	0.39394	0.62854	0.04113
Bd5	0.00216	0.005	0.00216	0.00216	1	0.00216	0.00216	0.00216	0.00477	0.005	0.00216	0.005	0.00216
Md1	0.00216	0.005	0.04113	0.00639	0.00216	1	0.01515	0.00216	0.04963	0.005	0.07765	0.02002	0.00216
Md2	0.00216	0.005	0.74749	0.80953	0.00216	0.01515	1	0.02597	0.00477	0.005	0.74835	0.47039	0.07765
Md3	0.00866	0.03671	0.00866	0.00866	0.00216	0.00216	0.02597	1	0.00477	0.005	0.17965	0.01291	0.33582
Md5	0.00477	0.0047	0.00477	0.00477	0.00477	0.04963	0.00477	0.00477	1	0.0047	0.00603	0.0047	0.00477
MBd1	0.17273	0.03006	0.005	0.005	0.005	0.005	0.005	0.005	0.0047	1	0.005	0.00492	0.03035
MBd2	0.00216	0.005	0.42253	0.39394	0.00216	0.07765	0.74835	0.17965	0.00603	0.005	1	0.29711	0.10869
MBd3	0.005	0.00492	0.62975	0.62854	0.005	0.02002	0.47039	0.01291	0.0047	0.00492	0.29711	1	0.04495
MBd5	0.33582	0.52038	0.04113	0.04113	0.00216	0.00216	0.07765	0.33582	0.00477	0.03035	0.10869	0.04495	1
